# A novel active biopolymer coating of pectin, potato starch, and pyrogallol: Impact on postharvest quality of tomato (*Solanum lycopersicum* L.)

**DOI:** 10.1111/1750-3841.70179

**Published:** 2025-04-04

**Authors:** Aparna Ramadoss, Venkata Giridhar Poosarla, Shaik Sadiya, Nagaveni Shivshetty

**Affiliations:** ^1^ Department of Life Sciences, GITAM School of Science GITAM (Deemed to be University) Visakhapatnam Andhra Pradesh India

**Keywords:** active packaging, antioxidant property, mechanical properties, pectin–potato starch‐pyrogallol, shelf life

## Abstract

**Abstract:**

Recently, there has been an increasing interest in biodegradable films for extending food's shelf life. This study developed pectin–potato starch‐based films incorporating varying pyrogallol concentrations and evaluated shelf life their physical, antioxidant, mechanical, optical, antibacterial, structural, biodegradation, and shelf‐life properties. Among the tested films (F1, pectin; F2, pectin + potato starch; F3, pectin + potato starch + 0.5%pyrogallol; and F4, pectin + potato starch + 1%pyrogallol), F4 exhibited superior antibacterial activity against *Staphylococcus aureus* (42 mm), *Klebsiella pneumoniae* (20.5 mm), and *Escherichia coli* (25.5 mm), antioxidant activity (AA) (95% (diphenylpicrylhydrazyl), 76% (metal chelating activity), and 87% (hydroxyl radical scavenging assay)), mechanical, and soil biodegradation. Fourier transform infrared spectroscopy and field emission scanning electron microscopy confirmed biocompatibility, whereas differential scanning calorimetry studies showed thermal stability. Shelf‐life studies on tomatoes at 30°C demonstrated that F4 film coating extended shelf life to 21 days by reducing weight loss (14.5%), total phenolic content (25 mg/100 g), AA (53.5%), firmness (46 N), and titratable acidity (0.38%) while maintaining the total soluble solids, pH, lycopene content, color, and microbial inhibition. This study introduces a novel active biodegradable film with enhanced antimicrobial, mechanical, and antioxidant properties for sustainable food packaging applications.

**Practical Application:**

This study introduces an eco‐friendly biopolymer coating formulated to extend the shelf life of food by reducing spoilage and maintaining quality during storage. The coating is cost‐effective, easy to produce, and can be used for industrial‐scale applications by giving a sustainable alternative to synthetic packaging. It can provide consumers with long‐lasting produce by maintaining freshness, reducing food waste, and promoting environmentally conscious food preservation practices.

## 1 INTRODUCTION

Biodegradable packaging, introduced in 1980, is now in high demand due to environmental benefits. Unlike nonbiodegradable polymers, which are dense and accumulate in the environment, potentially harming living beings, biodegradable polymers decompose quickly through microbial action, breaking down into CO_2_, H_2_O, and biomass. Packaging materials have evolved to include plastics, glass, paper, and metals, offering protection, convenience, and food security benefits. However, the environmental demand for nonbiodegradable materials has increased the demand for sustainable alternatives (Dwivedi et al., 2019; Shaikh et al., 2021; Versino et al., 2023).

There are several categories of natural sustainable polymers, such as carbohydrates and proteins. Pectin, a complex polysaccharide, constitutes about 35% of primary cell walls in some plant species, such as citrus fruits and apple pomace (Manjula et al., 2024). It mainly comprises over 65% galacturonic acid linked at positions O‐1 and O‐4 (Wusigale et al., 2020). Pectin is valued in the food industry for its natural availability, distinct flavor, and thickening and gelling abilities. Pectin structure includes rhamnose‐rich regions that enhance molecular interactions and galactose‐rich regions that promote entangled structure formation (Chen et al., 2020). Pectin's significant gelling property makes it an essential ingredient in biodegradable film production, and the gelation mechanism primarily depends on its degree of esterification, which was influenced by intrinsic and extrinsic factors like ionic strength, pH, temperature, solute presence, degree of methylation, molecular weight, and charge distribution along the molecule's backbone (Lara‐Espinoza et al., 2018).

Starch molecules consist of amylose (helical and linear) and amylopectin (branched). Starch molecules swell and burst when heated, forming a network that increases viscosity by gelatinization (Marichelvam et al., 2019; Sanyang et al., 2018). Starch can be obtained from various sources, such as corn starch (Nordin et al., 2020), cassava starch (Méité et al., 2024), yam starch (Estrada‐Girón et al., 2024), and potato starch (Prakash & Immanuel, 2023). Potato starch is most widely used for its processability, transparency, and oxygen barrier properties (Wang et al., 2025).

Active packaging has emerged as a solution to food preservation challenges, either absorbing compounds like oxygen or releasing beneficial antioxidants into the food, extending its shelf life. Oxygen significantly contributes to food degradation through oxidation, affecting color, flavor, texture, odor, and nutritional value. Essential oils and antimicrobial agents such as oregano (Garavito et al., 2020), chestnut flowers (Liu et al., 2024), clove (do Nascimento et al., 2023), cinnamon (do Nascimento et al., 2023), thymol (Poosarla et al., 2024), pyrogallol (Gaikwad et al., 2017a), and chitosan (Il'ina et al., 2024) are commonly utilized as active components in fruit coating formulations to enhance shelf life and quality. However, incorporating pyrogallol as an active component in these coatings has not yet been explored. Pyrogallol is a phenolic compound found in oak, eucalyptus, and other hardwoods. Comprehensive studies have shown pyrogallol to be safe for human consumption (<28 mg/kg body weight) and food products (NCBI, 2025; Gaikwad et al., 2017b). It features a benzene ring linked to 1,2,3‐triol, with hydroxyl groups enabling hydrophilic interactions and forming non‐covalent hydrogen bonds (Promsorn & Harnkarnsujarit, 2022). Pyrogallol is rich in flavonoid content, absorbs oxygen from its surroundings, protects food from spoilage, and provides antioxidant, antimicrobial, and analgesic properties (Gaikwad et al., 2017a; Uemura et al., 2016). Applying antimicrobial coatings on fruits and vegetables can significantly reduce postharvest losses (Garavito et al., 2020).

Tomato (*Solanum lycopersicum* L.) is a widely cultivated fruit globally (170 million tonnes per annum) (Conti et al., 2023). The tomatoes are stored at 10–15°C and 80%–90% relative humidity to extend the shelf life. Approximately 20%–42% of the tomato's postharvest losses worldwide were due to improper handling, transportation, and storage (Mohan et al., 2023; Sarma, 2018). In India alone, the majority of the produce is lost on the farm (15%), retail (12%), market, and during transportation stages due to pests, diseases, mechanical injury, and high rate of ripening (climacteric nature) (Lamba et al., 2024). It suggests a need to discover a better packaging material that extends tomatoes’ shelf life.

Several studies have been conducted on potato starch (Bhatia et al., 2025; Prakash & Immanuel, 2023; Sani et al., 2021), pectin (Agostinho & Rocha‐Filho, 2024; Cao et al., 2024; J. Wang et al., 2025), and pyrogallol (Gaikwad et al., 2024; Gaikwad et al., 2017a; Singh et al., 2019), but no report has been found on combining these three to form an active packaging. This study focuses on adding functional properties to the developed films, which would provide enhanced antibacterial and antioxidant properties (Aitboulahsen et al., 2020; Homthawornchoo et al., 2022; Liu et al., 2022; Song et al., 2023). Furthermore, limited research has explored the impact of incorporating pyrogallol into biodegradable films for shelf‐life analysis (Gaikwad et al., 2024; Singh et al., 2019). Addressing this gap may advance the field of sustainable packaging and align with Sustainable Development Goal 2 objective (Zero Hunger) by potentially enhancing food preservation and reducing waste. This study produced biodegradable active packaging by impregnating pyrogallol in pectin and potato starch and showed the extension of the shelf life of tomatoes using this conjugate, highlighting its sustainable packaging solutions.

## 2 MATERIALS AND METHODS

### 2.1 Chemicals and reagents

Pectin and 2,2‐diphenyl‐1‐picrylhydrazyl (DPPH) (Analytical grade (Sisco Research Laboratory)), pyrogallol (Fisher Scientific), Mueller Hinton agar (HIMEDIA), and glycerol (Qualigens) were purchased. Potato starch was extracted in the laboratory.

### 2.2 Extraction of potato starch

Fresh potatoes (*Solanum tuberosum*) were purchased from the local market of Visakhapatnam, India. They were adequately washed, peeled, chopped into small pieces, and blended with distilled water (DW) to form a puree. The puree was filtered with the help of a fine muslin cloth, and this filtered liquid was let to sediment for 6–8 h. Later, the supernatant was decantated to obtain white sediment (starch), which was left to dry in an incubator for 10–12 h at 37°C. The mass in the muslin cloth was washed 4–5 times for maximum extraction (Sani et al., 2021).

### 2.3 Film preparation

**FIGURE 1:**
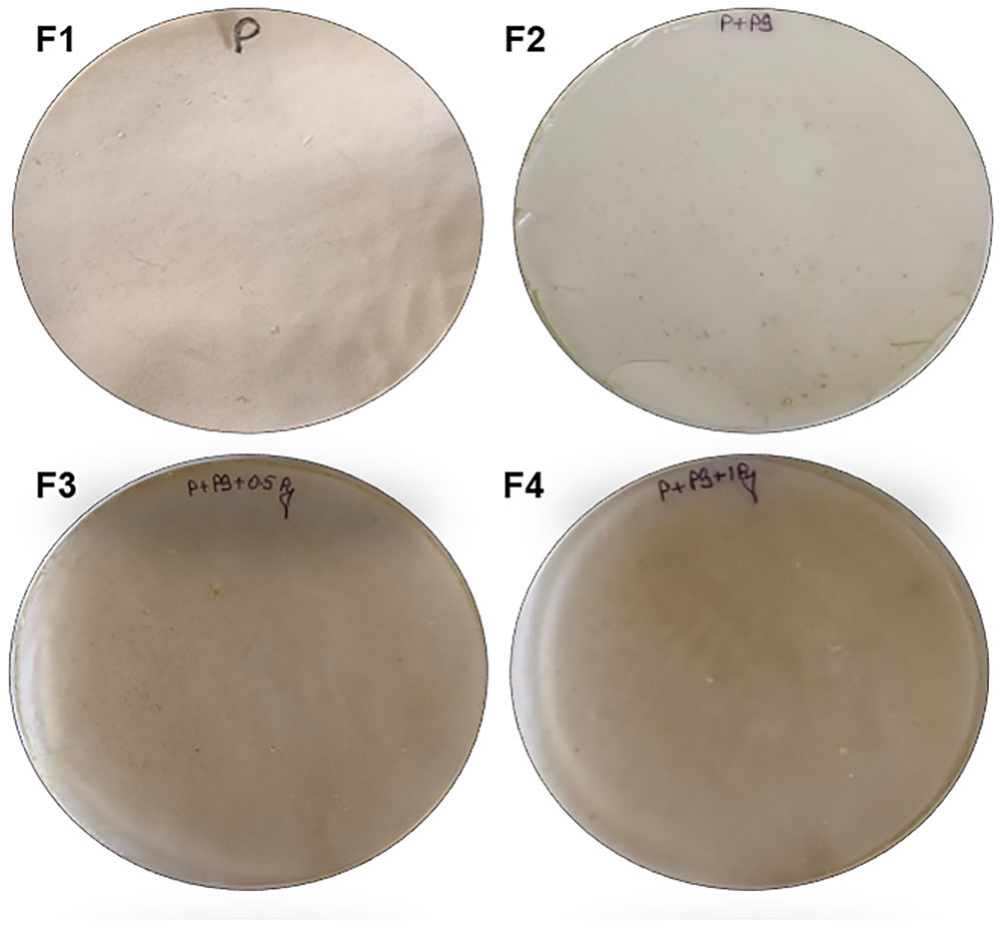
Development of biodegradable packaging films. F1, pectin; F2, pectin + potato starch; F3, pectin + potato starch + 0.5%pyrogallol; and F4, pectin + potato starch + 1%pyrogallol.

The film blends of pectin, potato starch, and pyrogallol were prepared using a solvent‐casting technique (Lal et al., 2021; Nisar et al., 2018). Pectin (4 g) and potato starch (0.5 g) were dissolved in DW, and the solution was made up to 100 mL. The formulated solution was heated at 80°C for 40–50 min till the solution was gelatinized completely on a hot plate with constant stirring. The film‐forming solution was cooled to room temperature at 30°C, and the glycerol (1%) was added as a plasticizer. Pyrogallol (0.5% and 1%) was added to the obtained solution. This solution was poured into a Petri plate (145 mm diameter) and was allowed to dry at 30°C for 24 h inside a biological oxygen demand incubator (Model‐925100, Labocare). Once dried, the films were peeled off carefully and stored in an incubator at 35–37°C. The films prepared were labeled F1, pectin; F2, pectin + potato starch; F3, pectin + potato starch + 0.5%pyrogallol; and F4, pectin + potato starch + 1%pyrogallol (Figure 1). These combinations were finalized after attempting multiple formulations with pectin and potato starch and are displayed in Figure S1.

### 2.4 Characterization of the composite films

#### 2.4.1 Physical properties of the film

##### 2.4.1.1 Moisture content

The films were cut into 2 × 2 cm^2^ and dried at 105°C for 24 h to determine the moisture content. Then, it was calculated by measuring the percentage of dry residue (Poosarla et al., 2024). The moisture content for each film was estimated in triplicates according to the following equation:

(1) $$\mathrm{Moisture}\;\mathrm{content}\;\left(\%\right)=\frac{W_{1}-W_{2}}{W_{1}}\times 100$$

where *W*_1_ is the initial weight, and *W*_2_ is the weight after drying.

##### 2.4.1.2 Water solubility

The film samples were cut into 2 × 2 cm^2^ and were dried in a hot air oven. The dried film weight was noted down. They were immersed in 10 mL DW and were placed at 25°C for 24 h. These films are centrifuged and re‐dried in the hot air oven for their final weight at 105°C for 24 h (Poosarla et al., 2024). The water solubility was calculated according to the following equation:

(2) $$\mathrm{Water}\;\mathrm{solubility}\;\left(\%\right)=\frac{W_{i}-\;W_{f}}{W_{i}}\times 100$$

where *W_i_* is the initial dry weight of the film, and *W_f_* is the final dry weight of the film.

##### 2.4.1.3 Water vapor permeability (WVP)

The WVP of the films was measured to evaluate the rate at which water diffuses through the composite film over a specific period. The films were sealed on a test tube filled with anhydrous calcium chloride. These test tubes were kept in a desiccator saturated with sodium chloride solution at 30°C. WVP was measured every 24 h at 75% relative humidity until the weight change was approximately 0.001 g (Poosarla et al., 2024). The WVP was determined by the following equation:

(3) $$\mathrm{Water}\;\mathrm{vapor}\;\mathrm{permeability}\;\left(g/\left(m\;s\;\mathrm{Pa}\right)\right)=\frac{\left(m1-m0\right)\;L}{At\Delta P}$$

where *m*1 is the final weight of the test tube, g; *m*0 is the initial weight of the test tube, g; *L* is the thickness of the film, m; *A* is the exposed area of the film, m^2^; *t* is the time, s; and Δ*P* is the water vapor pressure on both sides of the film, Pa.

##### 2.4.1.4 Water activity

The sample films were placed in a water activity meter (LabSwift‐aw, Novasina) and calibrated at 30°C using suitable standards. The water activity of the developed films was measured in triplicates for each film (Poosarla et al., 2024).

#### 2.4.2 Antioxidant activity

##### Diphenylpicrylhydrazyl (DPPH) assay

DPPH assay was used to measure the antioxidant activity (AA) of developed films. DPPH is a free radical scavenging assay where 4 mg of DPPH was dissolved in 100 mL methanol to form the solution. A sample film of 6 mg was added to 3 mL of DPPH solution and kept in the dark at room temperature. Later, the absorbance was measured at 517 nm (Poosarla et al., 2024). The film's AA was calculated using the following equation:

(4) $$\mathrm{DPPH}\;\mathrm{assay}\left(\%\right)=\frac{A_{0}-A_{T}}{A_{0}}\times 100$$

where *A*_0_ is the absorbance value for the control film, and *A_T_* is the absorbance value for the sample solution of the film.

##### Metal chelating activity (MCA)

The MCA was determined by mixing 1 mL of the film‐forming solution with 3.7 mL DW. A concentration of 2 mM FeCl_2_ (0.1 mL) and 5 mM 3‐(2‐pyridyl)‐5,6‐bis(4‐phenyl‐sulfonic acid)‐1,2,4‐triazine (ferrozine) (0.2 mL) were added and incubated for 20 min. The absorbance was measured at 562 nm using a spectrophotometer (Shimadzu UV 1800 spectrophotometer), and the chelating activity was calculated using the following equation using 1 mL DW as control (Poosarla et al., 2024):

(5) $$\begin{array}{ccc} & & \mathrm{Metal}\;\mathrm{chelating}\;\mathrm{activity}\;\left(\%\right)\\ & & =1-\frac{\mathrm{Absorbance}\;\mathrm{of}\;\mathrm{sample}\;\mathrm{at}\;562\;\mathrm{nm}}{\mathrm{Absorbance}\;\mathrm{of}\;\mathrm{control}\;\mathrm{at}\;562\;\mathrm{nm}}\times 100 \end{array}$$

##### Hydroxyl radical‐scavenging activity (HRSA)

To assess HRSA, film solution (1.5 mg/mL) was mixed with 3 mM ferrous sulfate (FeSO_4_) (100 µL), 3 mM 1,10‐phenanthroline dissolved in 7.4 pH 0.1 M phosphate buffer (100 µL), and 0.01% hydrogen peroxide (100 µL). This mixture was incubated for 1 h at 37°C, and the absorbance was measured at 536 nm using a spectrophotometer (Poosarla et al., 2024). The following equation was used to calculate the results:

(6) $$\begin{array}{ccc} & & \mathrm{Hydroxyl}\;\mathrm{radical}\;\mathrm{scavenging}\;\mathrm{activity}\;\left(\%\right)\\ & & =0.5\times \frac{A_{S}-A_{1\;}}{A_{0}-A_{1\;}}\times 100 \end{array}$$

where *A_S_* is the absorbance of the sample, *A*_1_ is the absorbance of the control solution containing 1,10‐phenanthroline, FeSO_4_, and H_2_O_2_, and *A*_0_ is the absorbance of the blank solution containing 1,10‐phenanthroline and FeSO_4_.

#### 2.4.3 Mechanical properties of the films

The film samples’ thickness, density, tensile strength (TS), elongation at break (EAB), and Young's modulus (YM) were evaluated using a texture analyzer using a rectangular specimen (70 × 15 mm^2^) (Texture Analyzer‐MX2‐500N, Imada) according to ASTM standard D882‐02 (Poosarla et al., 2024). The thickness of the film was measured using a vernier calliper (Mitutoyo Absolute, CD‐6″CSX); density was calculated by determining the film's weight, area, and thickness. Film shrinkage was measured accordingly (Oliveira, 2018). Each parameter was measured in triplicates for each developed film.

#### 2.4.4 Optical properties of the film

##### 2.4.4.1 Transparency and opacity

Transparency and opacity were measured by cutting the films into 4 × 1 cm^2^ rectangular strips. A UV spectrophotometer was employed to measure the film transmittance at 800 nm. These strips were placed inside the spectrophotometer cell, and an empty cell was used for reference (Balakrishnan et al., 2018). The transmittance was calculated using Equation (7). The opacity was measured at 670 nm using Equation (8) (Poosarla et al., 2024):

(7) $$\mathrm{Transmittance}\;\left(\%\right)=\frac{T_{\mathrm{control}}-\;T_{\mathrm{film}}}{M_{t}}$$

where *T*_control_ is the OD of the blank cuvette, *T*_film_ is the OD of the cuvette with the film, and *M_t_* is the percentage of potato starch with respect to pectin mass:

(8) $$\mathrm{Opacity}\;\left(\%\right)=\frac{\mathrm{Ab}s_{670}}{x}$$

where Abs_670_ is absorbance at 670 nm, and *x* is the thickness of the film (mm).

##### 2.4.4.2 Film color

The color of the developed film was measured using HunterLab Colorimeter (ColorFlex EZ). The different *L*, *a*, and *b* readings were recorded for each film. *L* depicts the whiteness (100) or darkness (0), +*a* (red) to −*a* (green), and +*b* (yellow) to −*b* (blue) indices. The colorimeter was calibrated using a white standard calibration plate (L* = 93.71, a* = −1.27, and b* = 0.50). The color difference (∆*E*) (Bharti et al., 2022) was measured using Equation (9), and the whiteness index (WI) (Prachayawarakorn & Poomkaew, 2020) was measured using Equation (10). The values were expressed according to the International Commission on Illumination (CIE). Each parameter was measured in triplicates for each developed film:

(9) $$\Delta E=\sqrt{{\left(L^{*}-L\right)}^{2}+{\left(a^{*}-a\right)}^{2}+{\left(b^{*}-b\right)}^{2}}$$

(10) $$\mathrm{WI}=100-{\left[{\left(100-L\right)}^{2}+\left(a^{2}+b^{2}\right)\right]}^{\frac{1}{2}}$$

#### 2.4.5 Antibacterial activity of the prepared films

The antibacterial activity of the prepared films was evaluated using Kirby Bauer's disc diffusion method (Poosarla et al., 2024). Fresh broth cultures of *Escherichia coli* ATCC 25922, *Staphylococcus aureus* ATCC 25923, and *Klebsiella pneumoniae* ATCC 13883 were prepared, which were 24 h old. Before inoculation, the inoculum was standardized using McFarland standards (0.5 = 1.5 × 10^8^ CFU/mL). The Mueller Hinton agar plates were inoculated using a cotton swab with the respective cultures (*E. coli*, *S. aureus*, and *K. pneumoniae*). The films were cut in a 5 mm radius and were placed on the agar plates. The inoculated agar plates were incubated at 37°C temperature for 24 h, and the diameter of the inhibition zone was measured in mm. The antibacterial activity was also performed against antibiotic disc (amoxicillin) and pyrogallol alone as controls. All these experiments were performed in triplicates.

#### 2.4.6 Fourier transform infrared (FTIR) analysis

A Bruker Alpha‐II ATR‐FTIR (attenuated total reference‐Fourier transform infrared spectrophotometer) was used to investigate the intermolecular interactions of the film's matrix. The instrument featured a high‐resolution deuterated triglycine sulfate detector and zinc selenide (ZnSe) beam splitter. Spectra were collected in the 4000–500 cm^−1^ range with a scanning resolution of 4 cm^−1^ and 80 scans per sample average. The film sample was analyzed under the Fourier transform infrared (FTIR) probe, and data were processed using OPUS 7.1 software (Poosarla et al., 2024).

#### 2.4.7 X‐ray diffraction (XRD) analysis

A powder x‐ray diffraction (XRD) device (Bruker D8 Advance, USA) was used to record the data at 40 mA current with Cu *Kλ* (*λ* = 1.5406 Å) and a voltage of 40 kV. The samples are measured in the 2*θ* 05°–80° range with 0.02° increment at 2 s/step. This measured the degree of crystallinity of potato starch, pectin, and pyrogallol (Rao et al., 2024).

#### 2.4.8 Differential scanning calorimetry (DSC) analysis

The thermal properties of the polymer membranes were characterized using a differential scanning calorimetry (DSC) analyzer (Shimadzu DSC‐60) according to the D3418‐08 ASTM standard. A 10 mg sample from each film was placed in the sample pan of DSC equipment, and an aluminum pan was used as a reference. Each sample was heated from 25 to 250°C, at a rate of 5°C/min with a nitrogen atmosphere.

#### 2.4.9 Morphological analysis

The microstructure of the polymer cross‐section was observed using field emission scanning electron microscopy (FE‐SEM) (Tescan, Mira) to determine the surface porosity. A small section of the film composite was cut, and then 10 nm gold was plated in an argon atmosphere using a LUXOR metal coater. The films were attached using a double‐sided carbon tape, and 3 keV accelerating voltage was used to examine the film samples (Xie et al., 2021).

#### 2.4.10 Soil biodegradability of the developed films

Biodegradation is a crucial parameter that helps assess a product's shelf life. Biodegradation of the films was performed using red soil. About 3 × 2 cm^2^ length films were placed inside mesh bags to identify easily, and they were kept inside the soil at room temperature (30°C). Water was sprinkled on the soil regularly to maintain moisture, and the changes were monitored (Poosarla et al., 2024). This parameter was performed in triplicates, and the results were calculated using Equation (11). Before and after soil biodegradation, the fabricated films were compared to observe their microstructure differences by FE‐SEM:

(11) $$\mathrm{Biodegradation}\;\mathrm{percentage}=\frac{W_{i}-W_{f}}{W_{i}}\times 100$$

where *W_i_* is the initial weight of the film before the process of biodegradation starts, and *W_f_* is the final weight of the film once the process of biodegradation has started.

### 2.5 Shelf‐life studies

Shelf life is an important parameter that should be studied to determine the number of days a product is in edible condition. The tomato was chosen for this parameter due to its high respiration rate as an excellent climacteric fruit. Moreover, tomatoes are regularly consumed worldwide and have a relatively low shelf life.

#### 2.5.1 Formulation of coating

Coatings were formulated accordingly with F1, pectin; F2, pectin + potato starch; F3, pectin + potato starch + 0.5%pyrogallol; and F4, pectin + potato starch + 1%pyrogallol.

#### 2.5.2 Application of coatings on tomatoes

Fresh tomatoes were brought from the local markets of Visakhapatnam, Andhra Pradesh, India, on March 17, 2024. They were transferred in a ventilated car to the laboratory. The tomatoes were sorted according to shape, size, and color, free from defects, and with good physical appearance and integrity. The tomatoes were washed with 2% sodium hypochlorite solution, followed by DW. The apparatus coming in contact with the tomatoes was thoroughly sanitized before conducting any experiments.

The formulated coatings were carefully applied by dipping the tomatoes. Two hundred and forty tomatoes were randomly grouped into uncoated and coated tomatoes (pectin (4%) (F1), potato starch (0.5%) (F2), pyrogallol (0.5% (F3) and 1% (F4)). The samples were dipped in the composites for 2 min and then air‐dried at 30°C for 15 min. This whole method was repeated two more times for even coating. They were then dried for 3 h and stored at 30°C (Poosarla et al., 2024). This experiment was performed in triplicates.

These uncoated and coated tomatoes underwent tests for several parameters for 21 days, and the analysis was performed at intervals of every 3 days. Parameters, including weight loss rate, total soluble solids (TSS), pH, titratable acidity (TA), total phenolic content (TPC), AA, firmness, and lycopene content, were performed accordingly (Poosarla et al., 2024). Weight loss rates were monitored using a precision balance (Venchal Scientific), TSS using a digital refractometer (ATAGO), and pH was measured using a digital pH meter (ESICO 1010). The L*, a*, b*, and the ratio of red and yellow (a*/b*) of the tomatoes were measured using a HunterLab Colorimeter (González‐Casado et al., 2018). To measure firmness, a 2 mm radius cylindrical probe (TA‐AACC36) is used with a 1 mm/s pretest speed, 2 mm/s test speed, 50% compression pressure, 15 s waiting time, 0.08 N trigger force, 7.0 mm deformation, and 1.0 mm/s posttest speed. For each test, tomatoes were used only once as the tomato samples were destructive.

#### 2.5.3 Microbial analysis

The microbial analysis was performed by counting the total mesophilic aerobic bacteria, yeast, and mold (Salas‐Méndez et al., 2019). The tomato puree (coated and uncoated) was mixed with 0.1% sterilized peptone water in a ratio of 1:9 and was homogenized for 2 min. Serial dilution of each sample was performed from the homogenized sample. For mesophilic aerobic bacteria, autoclaved molten nutrient agar (NA) was used, and yeast extract peptone dextrose (YEPD) agar medium was used for yeast and mold detection. Each dilution of 1 mL (1 × 10^−2^ to 1 × 10^−5^) was poured in a sterile Petri plate. NA plate was left for 2 days at 37°C and YEPD agar plate for 5 days at 25°C. The results were expressed as the logarithm of colony‐forming units per gram (log CFU/g).

### 2.6 Statistics

One‐way ANOVA (analysis of variables) followed by Tukey's honestly significant difference and two‐way ANOVA followed by the Bonferroni test were conducted using the software OriginPro 8.5 to test the differences between the variances. Pearson correlation was utilized for loadings (physical, antioxidant, mechanical, optical, film color, and antimicrobial). Data were considered significant at *p* < 0.05.

## 3 RESULTS AND DISCUSSION

### 3.1 Thickness and shrinkage

The incorporation of potato starch and pyrogallol into pectin increased the film's thickness from 0.23 mm (F1) to 0.32 mm (F4) (Table 1). This is due to the presence of components like starch, pectin, and phenols, which influence the thickness of the film as they embed into the film matrix and increase the thickness of the film (Sganzerla et al., 2020). Similar results of the increase in film thickness were observed when gelatin and chitosan (control) were blended with *Ziziphora clinopodioides* essential oil and ethanolic red grape seed extract, where the thickness of the film increased from 0.05 mm in the control film to 0.08 mm (Shahbazi, 2017). F4 film showed maximum shrinkage compared to the other films (data shown in Table S1). This happens due to the increase of hydrophilic components in the film matrix as hydrophilic molecules, such as pyrogallol, absorb more moisture. The absorbed moisture content decreases during drying or heating, causing the film matrix to contract and shrink. Additional hydrogen bonds form, resulting in size reduction and leading to shrinkage (Sutanto et al., 2019; Zamruddin et al., 2025).

**TABLE 1:** Physical properties of the developed films.

Films	*T* (mm)	MC (%)	WS (%)	WVP (10^−11^ g/(m s Pa))	*D* (g/cm^3^)	WA	Ty (%)	*O* (A/mm)	AA (%)		
									DPPH	HRSA	MCA
F1	0.23 ± 0.01^c^	24 ± 1^a^	93 ± 1.5^a^	3.11 ± 0.16^c^	0.12 ± 0.01^c^	0.63 ± 0.01^b^	87.5 ± 0.5^a^	0.28 ± 0.001^d^	18 ± 2^c^	27.5 ± 0.7^c^	12 ± 0.6^c^
F2	0.24 ± 0.01^c^	22 ± 1.5^a^	92 ± 1.5^a^	3.35 ± 0.16^c^	0.13 ± 0.02^c^	0.63 ± 0.01^b^	78.6 ± 0.3^b^	0.44 ± 0.001^c^	23 ± 1^b^	29.6 ± 0.7^c^	14 ± 1^c^
F3	0.27 ± 0.01^b^	18 ± 1^b^	92 ± 1^a^	4.39 ± 0.13^b^	0.15 ± 0.01^b^	0.65 ± 0.01^ab^	60.9 ± 0.9^c^	1.1 ± 0.001^b^	93 ± 1^a^	71.5 ± 1^b^	59 ± 1^b^
F4	0.32 ± 0.02^a^	16 ± 1^b^	91 ± 1^a^	5.43 ± 0.38^a^	0.19 ± 0.01^a^	0.66 ± 0.01^a^	56.1 ± 0.3^d^	1.13 ± 0.001^a^	95 ± 1.5^a^	87.3 ± 1.4^a^	76 ± 2^a^

*Note*: F1, pectin; F2, pectin + potato starch; F3, pectin + potato starch + 0.5%pyrogallol; and F4, pectin + potato starch + 1%pyrogallol. All data are averages of three measurements and reported as mean ± standard deviation (SD). Different letters (a, b, c, d) indicate significant differences between groups at *p* < 0.05 according to Tukey's honestly significant difference (HSD) test.

Abbreviations: AA, antioxidant activity; *D*, density; DPPH, diphenylpicrylhydrazyl assay; HRSA, hydroxyl radical‐scavenging activity; MC, moisture content; MCA, metal chelating activity; *O*, opacity; SI, swelling index; *T*, thickness; Ty, transparency; WA, water activity (*a*_w_); WS, water solubility; WVP, water vapor permeability.

### 3.2 Moisture content

A key property of a film is its water resistance, and pyrogallol in this study exhibits strong inter‐ and intramolecular OH―H and OH―O interactions and π–π stacking, contributing to the film's characteristic feature (Shin et al., 2019; Sutanto et al., 2019). The anhydrous pyrogallol was thermodynamically stable, and these interactions and the molecule's polarization occurred well. According to a study, water protons and pyrogallol position accelerated the dehydration mechanism (Braun et al., 2013). This reduces moisture content from 24% to 16% (Table 1). Additionally, glycerol's low molecular weight facilitates the linking of polymer chains (Aitboulahsen et al., 2020). A similar trend of a decrease in moisture content from 17% to 16% was observed in a study where the addition of an active component (5% lemon pomace extract) was added to guar gum (1%) (Giacondino et al., 2024).

### 3.3 Density

The density of the prepared films increases from 0.12 to 0.19 g/cm^3^ with the addition of pyrogallol (Table 1) due to the presence of phenolic components of the pyrogallol (Gaikwad et al., 2017a). The amylose in starch helps form huge crystallized regions, which are embedded by the phenolic components in the film's matrix to increase the density (Domene‐López et al., 2019). The results are in corroboration with an increase in density from 0.06 to 0.13 g/cm^3^ observed with the increase in the concentration of thymol from 0.3% to 5% when thymol was impregnated with pectin‐carboxymethyl cellulose‐polyhydroxybutyrate conjugate (Poosarla et al., 2024).

### 3.4 Water solubility

The formulated biodegradable films are water‐soluble because of hydrophilic components like pectin, potato starch, and pyrogallol. Pyrogallol is organically polar, with partially positive and negative charges at OH―H and OH―O atoms. The three OH groups are responsible for the formation of intermolecular hydrogen bonding (Sutanto et al., 2019). There is no significant change in the water solubility values (Table 1).

### 3.5 Water vapor permeability (WVP)

The WVP of the polymers increases with the addition of pyrogallol from 3.1 × 10^−11^ g/(m s Pa) (F1) to 5.4 × 10^−11^ g/(m s Pa) (F4). The presence of hydroxyl groups in the composite films was a key factor influencing the WVP in films. Pyrogallol is a hydrophilic molecule with three hydroxyl groups embedded within the polymer matrix. These functional groups interact with the polymer chains, allowing water vapor to pass more easily. They promote the adsorption of water molecules and enhance their movement through the film. As a result, pyrogallol‐containing films (F3 and F4) show higher WVP than pectin (F1) or pectin and potato starch (F2) films. A similar increasing trend of WVP was observed in a study where modified LDPE films were fabricated with various concentrations of pyrogallol (1%, 3%, 5%, 10%, and 20%) (Gaikwad et al., 2017a). Furthermore, the WVP of 5.4 × 10^−11^ g/(m s Pa) of our study is in close alignment with the WVP value of 7.9 × 10^−11^ g m/m^2^ s Pa for the film prepared with rice‐starch‐pectin blended with green tea extract (1%, w/v) (Homthawornchoo et al., 2022). In another study, a similar trend was observed with the increase in Ora‐pro‐nobis mucilage extracted from the leaves of *Pereskia aculeata* Miller (Cactaceae family) from 8.3 to 25.9 (g water mm/(day m^2^ kPa)) (Oliveira et al., 2019).

### 3.6 Transparency and opacity

Transparency and opacity are the two essential parameters for consumers’ acceptance of a film. The transparency and opacity are also affected in the pyrogallol films from 88% (F1) to 56% (F4) and 0.28 A/mm (F1) to 1.13 (F4) A/mm, respectively (Table 1) due to the phenolic compounds present in pyrogallol, which affect the opacity and color of the film. The films developed with pyrogallol (F3 and F4) are opaque due to pyrogallol's phenolic nature and color pigments. The control film (F1) was completely transparent and clear due to the coalescence of pectin. Potato starch contains amylopectin, which contains higher long chains, leading to chain reordering and forming a transparent starch matrix (Domene‐López et al., 2019). The results align with a study where the transparency decreased from 60% to 30% when pineapple leaf cellulose nanofibers concentration increased from 0 to 4 wt% in the potato‐based thermoplastic starch film (Balakrishnan et al., 2018). In another study, an increase in the opacity from 0.8 to 4.8 A/mm was observed, with an increase in young apple polyphenols from 0% to 1.5% in 2.75% pectin film (Nisar et al., 2019).

### 3.7 Water activity

Water activity is one of the most critical parameters in a biodegradable film as it determines the growth of microorganisms. The water activity of the pyrogallol films increased from 0.63 in film F1 to 0.66 in film F4 (Table 1) due to the polar nature of pyrogallol. The partial positive and negative charges are located at the OH–H and OH–O atoms in pyrogallol, and these charges, along with intermolecular hydrogen bonding, lead to a rise in the film's water activity (Sutanto et al., 2019).

### 3.8 Mechanical properties

The maximum stress a material can withstand while being stretched or pulled before breaking has been confirmed by a film's TS. Each film must exhibit a degree of flexibility to facilitate packaging operations. However, the film must also possess sufficient TS to resist tearing at handling conditions (Rech et al., 2021). The TS of the F2 (pectin + potato starch) was 4.3 MPa, and F4 was 4.5 MPa (Table 2). The results are in close accordance with another study where the TS for potato starch (5%) and apple peel pectin (3:1 ratio) film was 4.3 MPa, which increases to 4.7 MPa with the addition of microencapsulated *Zataria multiflora* essential oil (0.5%) (Sani et al., 2021). When plasticizers are added to films, the TS decreases, and the flexibility increases due to the internal hydrogen bonding, which increases the intermolecular space of the polymers. Hence, plasticizers reduce the TS and increase the EAB (Buso‐Rios et al., 2020; Rech et al., 2021). Potato starch helps increase TS due to the strong interaction between plasticizer and starch molecules that inhibit macromolecular movement. This was the possible reason for the increase in EAB and decrease in YM of the films that included potato starch (F2, F3, and F4) compared to the film that contained pectin alone (F1) (Domene‐López et al., 2019). F3 gave the best TS, possibly due to the higher interfacial cross‐linking density between film composites. However, F4 again contributed to a reduction in TS because of the increase in the phenolic hydroxyl group of pyrogallol (1%) (Qian et al., 2015). Along with the TS, the flexibility of F3 and F4 increases. The current study's findings showed superior TS than a previous study, where varying concentrations of thyme essential oil (10%, 20%, and 30% of dry corn starch mass) with corn starch (3%) showed a TS (2.1–3.4 MPa) and EAB (56.8%–23.5%), respectively (Cai et al., 2020). The stress–strain curve of the prepared formulation is demonstrated in Figure S2. The graph suggests that the F1 films are stiff, but the flexibility increases with the addition of an active component (pyrogallol (45%–71%)).

**TABLE 2:** Mechanical properties of the developed films.

Films	Mechanical properties		
	TS (MPa)	EAB (%)	YM (MPa)
F1	4.1 ± 0.1^c^	45 ± 2^c^	295 ± 3^a^
F2	4.3 ± 0.1^b^	61 ± 1^b^	275 ± 2^b^
F3	4.6 ± 0.2^a^	64 ± 1^b^	85 ± 2^c^
F4	4.5 ± 0.1^a^	71 ± 2^a^	60 ± 1^d^

*Note*: F1, pectin; F2, pectin + potato starch; F3, pectin + potato starch + 0.5%pyrogallol; and F4, pectin + potato starch + 1%pyrogallol. All data are averages of three measurements and reported as mean ± SD. Different letters (a, b, c, d) indicate significant differences between groups at *p* < 0.05 according to Tukey's HSD test.

Abbreviations: EAB, elongation at break; TS, tensile strength; YM, Young's modulus.

### 3.9 Antibacterial activity

**FIGURE 2:**
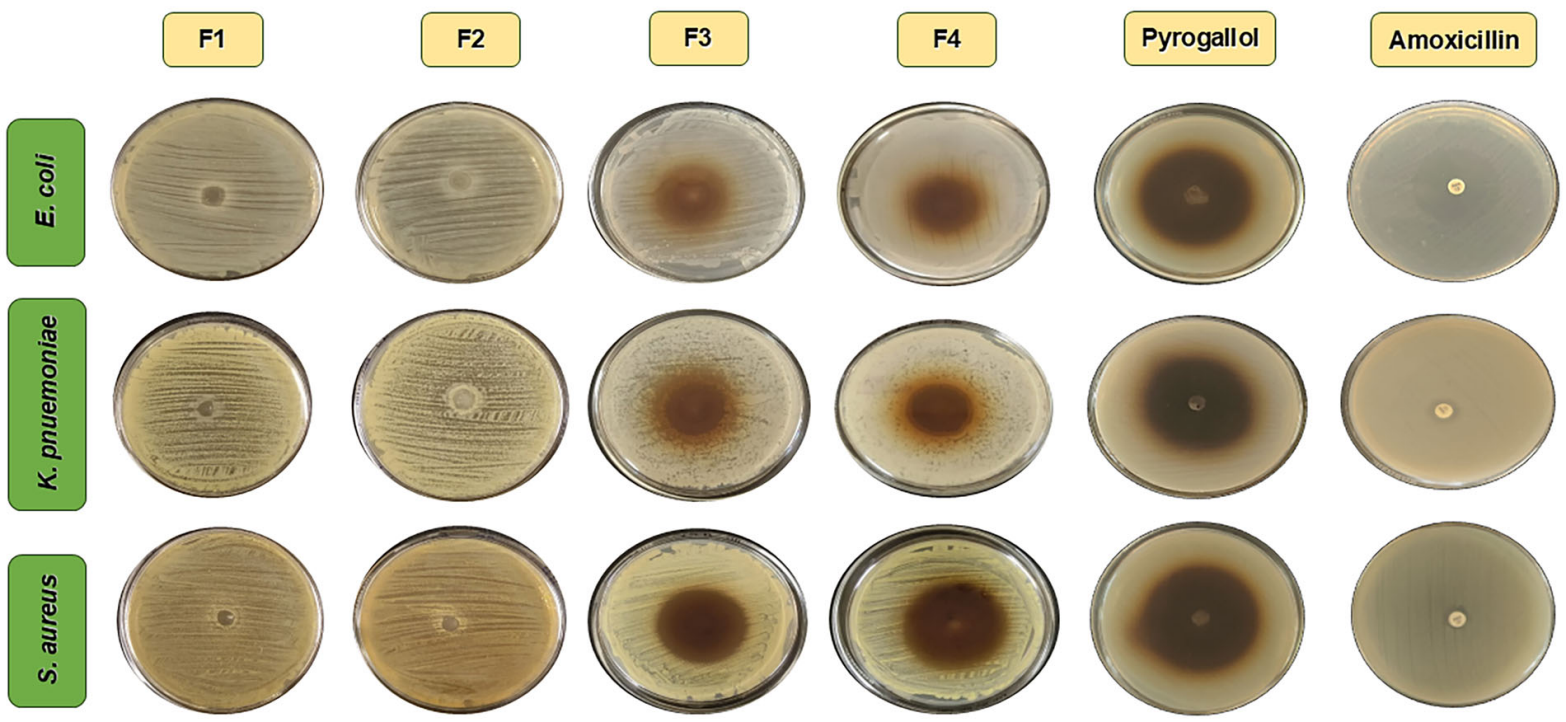
Antibacterial activity of developed films. F1, pectin; F2, pectin + potato starch; F3, pectin + potato starch + 0.5%pyrogallol; and F4, pectin + potato starch + 1%pyrogallol.

Antibacterial substances are those compounds that plants and animals produce to protect themselves from other microorganisms. No zone of inhibition (ZOI) was detected in the control films due to the absence of an antibacterial agent (pyrogallol). The films containing pyrogallol (F3 (pectin + potato starch + 0.5%pyrogallol) and F4 (pectin + potato starch + 1%pyrogallol)) had a visible ZOI. The largest zone was observed in F4 against *S. aureus* (42 mm) (Figure 2), followed by *E. coli* (25.5 mm) and *K. pneumoniae* (20.5 mm) (Table 3). Our results showed a bigger ZOI against *S. aureus* compared to *E. coli* and *K. pneumoniae*. Studies have demonstrated that pyrogallol penetrates easily through the cell wall of Gram‐positive bacteria and increases reactive oxygen species (ROS) sensitivity, whereas Gram‐negative bacteria have an additional outer membrane that limits pyrogallol's permeability (Oliveira et al., 2022). The size of the zone increases with the increase in the concentration of antimicrobial agents, that is, pyrogallol (Nisar et al., 2018). The results are in corroboration with an increase in the ZOI (21.7 mm for *S. aureus* and 18.7 mm for *E. coli*) with the increase in the amount of active component (0%–2.5% *Zanthoxylum armatum* DC. essential oil) in combination with film composition of konjac glucomannan (1%) and 0.6% chitosan (Wang et al., 2022). Similarly, a study composed of apple peel pectin, potato starch, zirconium oxide, and 0.25% (w/w of dry matter) *Z. multiflora* essential oil reported a ZOI of 21 mm against *E. coli* (Sani et al., 2021). In another study, with 0.5% *Mentha pulegium* essential oil and 1.5% *Lavandula angustifolia* essential oil, the ZOI against *S. aureus* was 21 and 18 mm, respectively (Aitboulahsen et al., 2020). The antibacterial activity against antibiotic disc (amoxicillin) and pyrogallol alone has been shown in Figure 2 and Table 3. The isolates were found in the resistant range of Clinical and Laboratory Standards Institute guidelines (Weinstein & Lewis, 2020).

**TABLE 3:** Antibacterial properties of the developed films.

Films	Antibacterial properties (mm)		
	*Staphylococcus aureus*	*Klebsiella pneumoniae*	*Escherichia coli*
F1	Not detected	Not detected	Not detected
F2	Not detected	Not detected	Not detected
F3	39 ± 0.5^c^	10 ± 1^c^	20.5 ± 0.5^c^
F4	42 ± 0.5^b^	20.5 ± 0.5^b^	25.5 ± 1^b^
Pure pyrogallol	45.5 ± 0.5^a^	33 ± 1^a^	37.5 ± 0.5^a^
Amoxicillin	9 ± 1^d^	10 ± 1^c^	19 ± 0.5^c^

*Note*: F1, pectin; F2, pectin + potato starch; F3, pectin + potato starch + 0.5%pyrogallol; and F4, pectin + potato starch + 1%pyrogallol. All data are averages of three measurements and reported as mean ± SD. Different letters (a, b, c, d) indicate significant differences between groups at *p* < 0.05 according to Tukey's HSD test.

### 3.10 Antioxidant activity

DPPH, MCA, and HRSA were used to calculate the antioxidant properties of the developed films. An increase in the oxygen scavenging property was observed from 18% to 95% DPPH, 12% to 76% MCA, and 27% to 87% HRSA in the films made of pyrogallol (F3 (pectin + potato starch + 0.5%pyrogallol and F4 (pectin + potato starch + 1%pyrogallol)) compared to the control film F1 (Table 1). Pyrogallol generates free radicals due to the reduction of one electron‐forming superoxide anion (O_2_^•−^), a precursor of most ROS (Madkour, 2019; Park, 2016). Pyrogallol shows high antioxidant properties as it irreversibly reacts with the singlet oxygen atoms to form pyrogallol quinone as it donates its electrons for scavenging the oxygen‐free radicals. Once the electrons are gained from pyrogallol, the unstable free radicals return to their normal ground state, eliminating the free radical (Gaikwad et al., 2017b). These results are in close agreement with several studies where pyrogallol‐capped silver nanoparticles (90 µg/mL) exhibited 80% AA (Sampath et al., 2021), and in another study when gelatin/chitosan films were impregnated with 1% lemon essential oil, which showed 60% AA (Tügen et al., 2020). Additionally, our study results corroborate with the films made of potato starch, pectin, and 1% juniper berry essential oil demonstrated 17% AA; potato starch, pectin, and 2% thyme essential oil microcapsules displayed an AA of 67% (Wang et al., 2025); and cationic potato peel starch and 5% curcumin exhibited 87% AA (Liu et al., 2022). Films F1 and F2 show less antioxidant properties than F3 and F4 (containing pyrogallol) due to the presence of galacturonic acid in pectin, which displays free radical scavenging (Yan et al., 2023).

### 3.11 Color measurement

The color measurement of the developed film was performed, and the films with low ∆*E* values were observed to be translucent and transparent. F1 had the highest WI (61.3) and the lowest ∆*E* (4.7%) values. With the addition of pyrogallol in F3 (pectin + potato starch + 0.5%pyrogallol) and F4 (pectin + potato starch + 1%pyrogallol), the WI decreases (54 (F3) and 52.7 (F4)) and ∆*E* increases (5% (F3) and 5.2% (F4)) (Table 4). The decrease in WI occurs due to the formation of quinones, which are oxidized products of phenols as they absorb light and oxygen, giving a black, dark brown, or yellowish appearance (Vercruysse et al., 2017). The findings of the current study are in close agreement with biodegradable films formed from tapioca starch‐carrageenan‐caraway (*Carum carvi* L.) essential oil (Bharti et al., 2022) and banana flour nanocomposite films blended with garlic essential oil (Orsuwan & Sothornvit, 2018).

**TABLE 4:** Color measurement of the developed films.

Films	*L*	*a*	*b*	∆*E* (%)	WI
F1	62.3 ± 0.022^a^	−0.3 ± 0.005^d^	8.8 ± 0.012^c^	4.7 ± 0.002^c^	61.3 ± 0.621^a^
F2	59.9 ± 0.012^b^	−0.1 ± 0.005^c^	10.1 ± 0.005^b^	4.8 ± 0.008^c^	58.7 ± 0.382^b^
F3	56 ± 0.005^c^	1.8 ± 0.005^b^	13.5 ± 0.009^a^	5 ± 0.005^b^	54 ± 0.243^c^
F4	53.8 ± 0.017^d^	2.3 ± 0.005^a^	13.8 ± 0.005^a^	5.2 ± 0.01^a^	52 ± 0.514^d^

*Note*: *L* indicates lightness; *a* indicates redness or greenness; *b* indicates yellowness or blueness; ∆*E*, color difference. F1, pectin; F2, pectin + potato starch; F3, pectin + potato starch + 0.5%pyrogallol; and F4, pectin + potato starch + 1%pyrogallol. All data are averages of three measurements and reported as mean ± SD. Different letters (a, b, c, d) indicate significant differences between groups at *p* < 0.05 according to Tukey's HSD test.

Abbreviation: WI, whiteness index.

### 3.12 Fourier transform infrared spectroscopy (FTIR)

**FIGURE 3:**
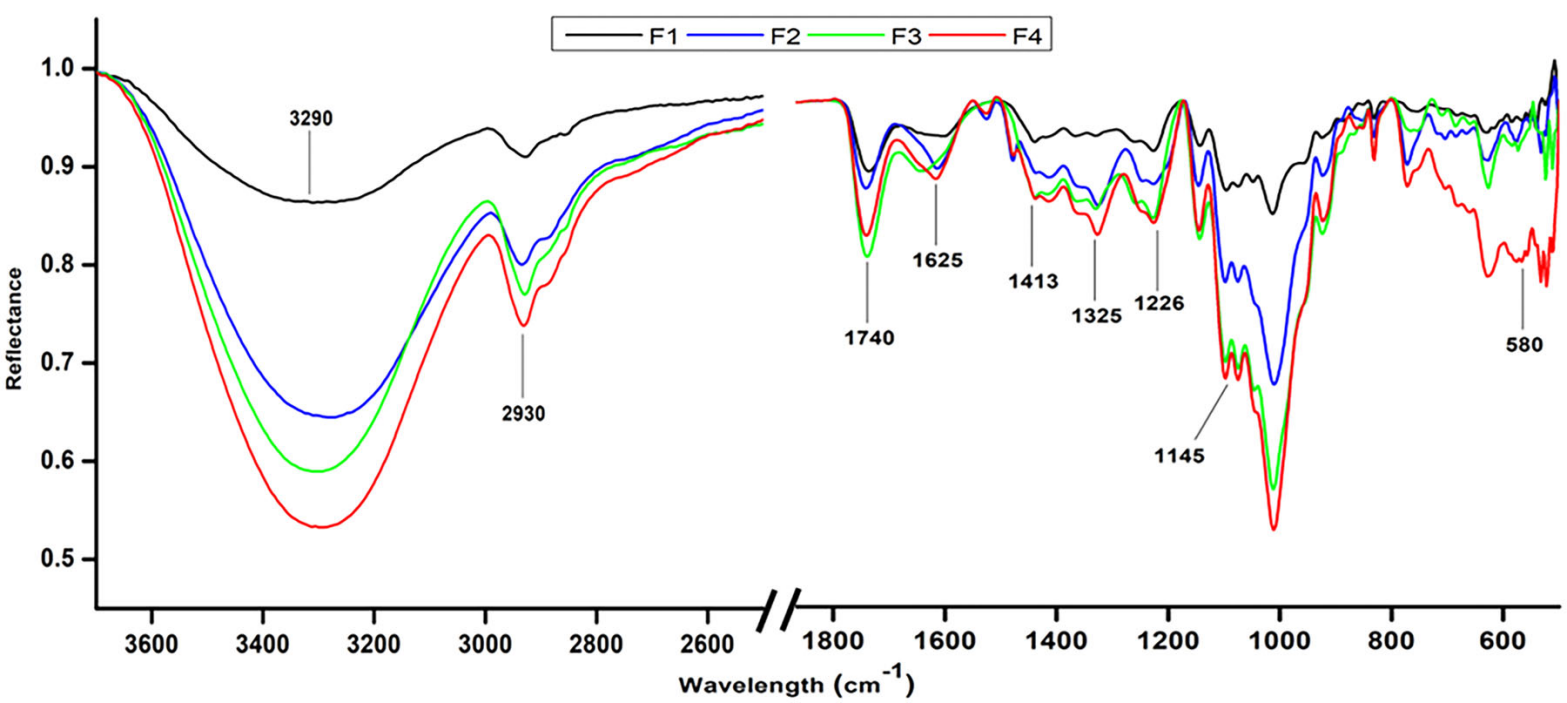
Fourier transform infrared (FTIR) spectra of the developed films. F1, pectin; F2, pectin + potato starch; F3, pectin + potato starch + 0.5%pyrogallol; and F4, pectin + potato starch + 1%pyrogallol.

The FTIR analysis of pectin, starch, and pyrogallol‐containing film revealed distinctive peaks indicative of the presence of these components (Figure 3). The observed peaks at wavenumbers 3274–3296 cm^−1^ correspond to the stretching vibrations of the OH group, which are characteristic of both pectin and starch due to their high OH content. The peaks at 2930–2934 cm^−1^ are also observed due to the stretching vibrations of aliphatic C―O and C―H bonds commonly found in starch molecules (Hebeish et al., 2009; Zhuang et al., 2023).

Furthermore, the presence of peaks at 1740–1741 cm^−1^ suggests the presence of carbonyl (C═O) stretching vibrations, which can be attributed to the ester functional groups in pectin molecules (Wathoni et al., 2019). Moreover, the peaks observed at 1325–1326, 1225–1226, and 1144–1145 cm^−1^ are associated with various functional groups in pectin and starch molecules, including C―O and C―N stretching vibrations (Hebeish et al., 2009; Wathoni et al., 2019).

The peaks at 1615–1631 and 1413–1414 cm^−1^ are consistent with the stretching vibrations of C═C bonds and bending vibrations of C‐H bonds, characteristic of unsaturated hydrocarbons such as pyrogallol. Lastly, the peak at wavenumber 520–771 cm^−1^ indicates the presence of bending vibrations of C―H and C―O bonds in the aromatic ring structure of pyrogallol (Ramasamy, 2016). Overall, the FTIR spectra provide valuable insights into the chemical composition and structure of the film samples, confirming the presence of pectin, potato starch, and pyrogallol based on the characteristic peaks observed.

### 3.13 X‐ray diffraction (XRD)

**FIGURE 4:**
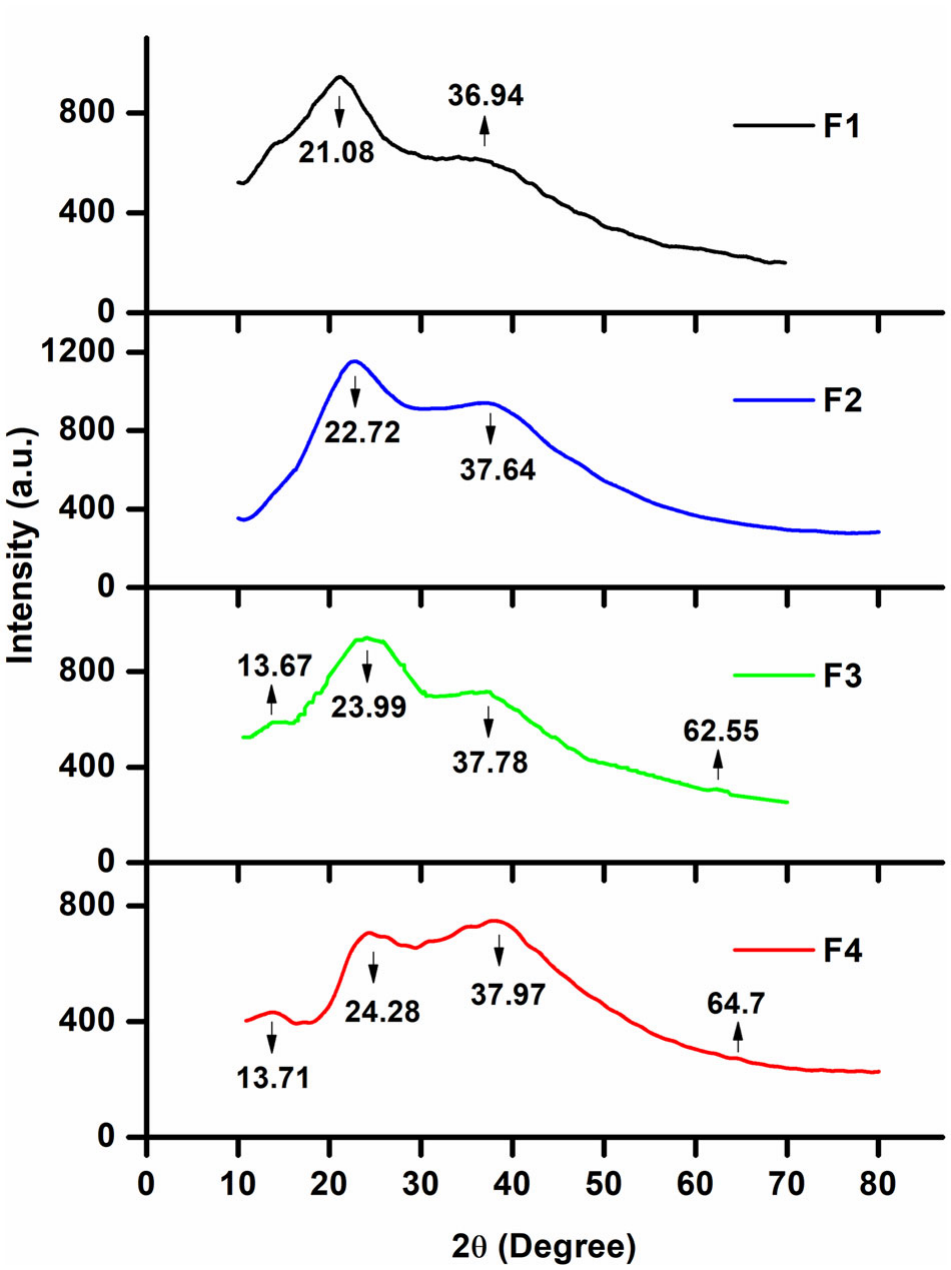
X‐ray diffraction (XRD) patterns of the prepared films. F1, pectin; F2, pectin + potato starch; F3, pectin + potato starch + 0.5%pyrogallol; and F4, pectin + potato starch + 1%pyrogallol.

XRD patterns were studied for the developed films where peaks of pectin and potato starch were observed at 21.08°–24.28° and 36.94°–37.97° (2*θ*) (Figure 4). Starch, in the presence of water, granular packing density, and amylopectin chain length, depicts different diffraction patterns. Cereal starches exhibit A‐type, high amylose maize, fruits, and tubers exhibit B‐type, and starches from legume seeds exhibit C‐type. Intense B‐type starch peaks were observed at 22.72°–24.28° (2*θ*). Peaks of pyrogallol were observed at 13.67°–13.71° and 62.55°–64.7° (2*θ*). These findings were in close agreement with other literature, such as for pectin (Mishra et al., 2011), starch (Meneguin et al., 2017), and pyrogallol (Kharouf et al., 2021; Thakuria et al., 2012). When the crystallinity of the developed films was calculated, it was observed that there was a steady rise in the crystallinity from F1 to F4. F1 demonstrated 25.5% crystallinity; F2, 29.4%; F3, 45.6%; and F4, 72.3%. This increasing trend suggests that the incorporation of potato starch and pyrogallol influenced the film's structure. Previous studies have demonstrated the semicrystalline structure of potato starch (Bertoft & Blennow, 2016) and the crystal structures of pyrogallol (Thakuria et al., 2012).

### 3.14 Differential scanning calorimetry (DSC)

**FIGURE 5:**
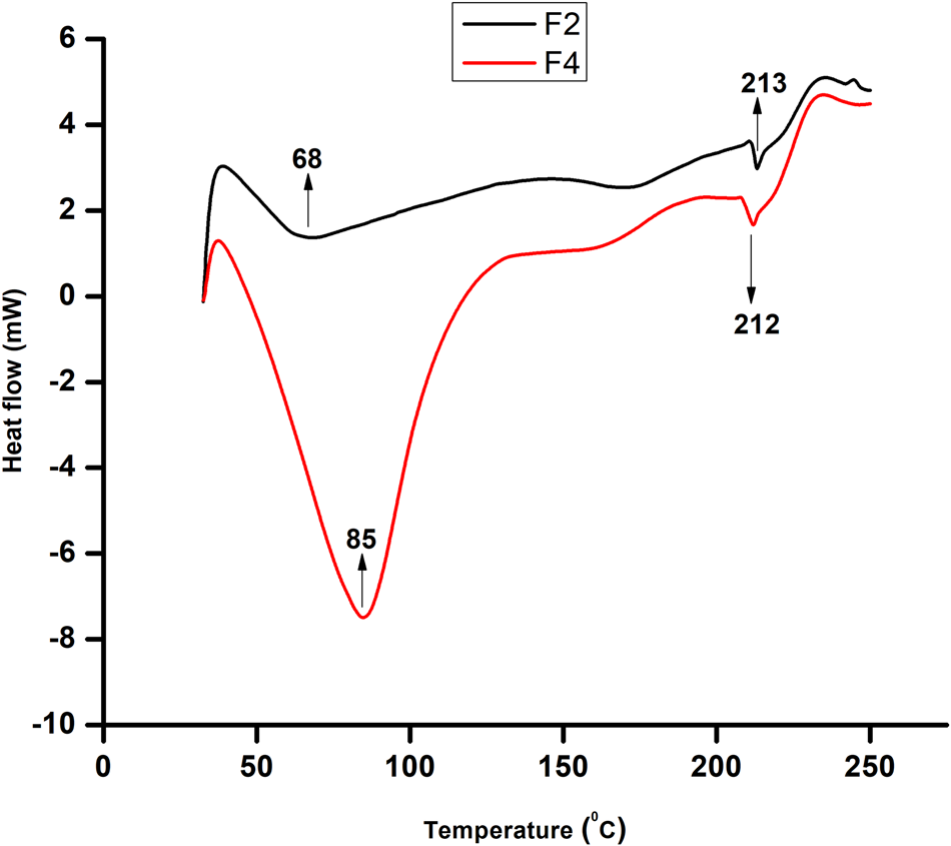
Differential scanning calorimetry (DSC) of the prepared films, F2, pectin + potato starch; and F4, pectin + potato starch + 1%pyrogallol.

The DSC analysis reveals several thermal transitions for the F4 and F2 films. The F4 film shows a sharp endothermic peak at 85°C, whereas the F2 film peaks at 68°C (Figure 5). This endothermic peak represents the glass transition temperature (*T*_g_), indicating the transition of the polymer from a hard or glassy state to a soft, rubbery state (LeBoeuf & Weber, 2000). The melting and denaturation of the polymer are observed at 213°C for F2 and 212°C for F4. This close similarity suggests that the presence of pyrogallol in the polymer matrix does not significantly alter the melting behavior of the films (Gaikwad et al., 2017a). A similar study showed a melting temperature of 107.52°C for films made of polyisocyanate, polyurethane, modified low‐density polyethylene, and pyrogallol (Gaikwad et al., 2017a).

### 3.15 Field emission scanning electron microscopy

**FIGURE 6:**
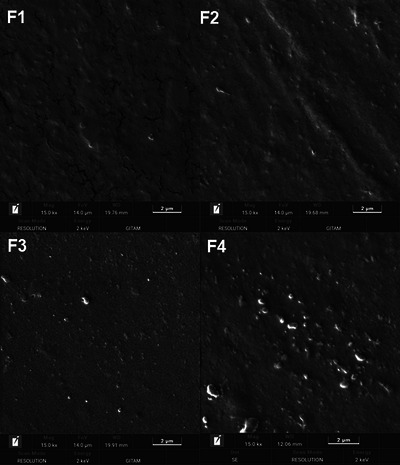
Field emission scanning electron microscopy (FE‐SEM) images of developed films. F1, pectin; F2, pectin + potato starch; F3, pectin + potato starch + 0.5%pyrogallol; and F4, pectin + potato starch + 1%pyrogallol.

The surface morphology of the F1, F2, F3, and F4 films was studied using FE‐SEM (Figure 6). This provides microstructural information about pectin, potato starch, and pyrogallol films with glycerol as a plasticizer. Potato starch acts as a filler, improves the plasticization effect, and fills the microcracks or voids in the polymer matrix of F2, F3, and F4 films (Gujral et al., 2021; Mendes et al., 2020; Resano‐Goizueta et al., 2018). Pectin and potato starch blends exhibit strong compatibility due to the extensive hydrogen bonding between the COOH and OH groups (Li et al., 2022; Yoon et al., 2006). The addition of pyrogallol leads to an increase in surface heterogenicity, leading to the formation of agglomerations in the film matrix. The size of the agglomerations increased with the increase in the concentration of pyrogallol. Similar findings were observed in other studies where thermoplastic cassava starch was blended with pyrogallol (Promsorn & Harnkarnsujarit, 2022) and poly(butylene adipate‐co‐terephthalate), poly(butylene succinate), and gallic acid blended films were fabricated (Pothinuch et al., 2024).

### 3.16 Soil biodegradation

**FIGURE 7:**
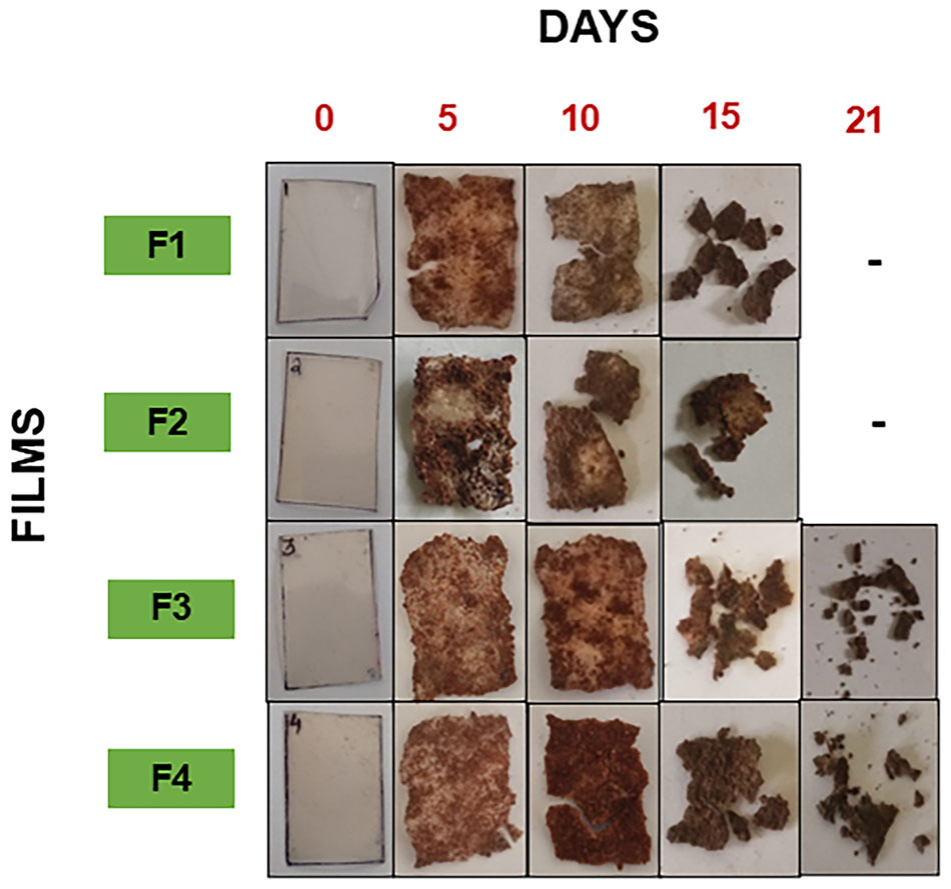
Soil biodegradation of the prepared films. F1, pectin; F2, pectin + potato starch; F3, pectin + potato starch + 0.5%pyrogallol; and F4, pectin + potato starch + 1%pyrogallol.

Biodegradation is a crucial parameter that helps assess a product's shelf life. Red soil was used for performing biodegradation. Several factors contribute to the process of biodegradation, such as temperature, pH, nutrients, oxygen, mineral content of the soil, film composition, and mesophilic microorganisms (Rech et al., 2020). Water was sprinkled on the soil to maintain the soil's moisture content every 24 h, and the films buried were observed for 21 days at 30°C (Figure 7). At the beginning of the biodegradation process, the films first absorbed the soil's moisture, leading to a rise in weight, and the films swelled up. However, the weight of the films again started to reduce due to the process of biodegradation. F1 (pectin) and F2 (pectin + potato starch) were entirely degraded within 21 days of soil biodegradation due to the micro void formation in the film matrix (Savenkova et al., 2000).

The remaining film samples (F3 and F4) were kept for further biodegradation. The films (F3 and F4) showed 88% and 82% biodegradation, respectively, because of the antimicrobial nature of the pyrogallol. Similar results of soil biodegradation of 70% were obtained in the case of starch‐based cinnamon essential oil films (He et al., 2021).

The SEM analysis (Figure S3) revealed distinct structural differences in the films, highlighting the impact of soil biodegradation. Films F2 and F4 exhibited significant morphological changes, characterized by increased surface roughness and the formation of cracks under soil burial conditions. These modifications are due to microbial activity, which progressively degraded the film matrix, highlighting the biodegradability of the developed films. It was also observed that the F4 film showed minimal surface unevenness, corrosion, and cracks, whereas the F2 film underwent complete fragmentation, leading to the surface unevenness and cracks due to the presence of the antibacterial agent pyrogallol thereby increasing the film's stability (de Souza et al., 2024; Oliveira et al., 2022). Similar results have been observed in a study where biodegradable films of corn starch, nanocellulose, and black tea extract have been fabricated (Malekzadeh et al., 2024).

### 3.17 Pearson's correlation

Based on the Pearson correlation analysis of the formulated films (F1–F4) (data shown in Table S2), significant relationships were observed among key physicochemical, mechanical, and functional properties. Film thickness exhibited a strong positive correlation with density and WVP; moisture content showed a strong negative correlation with thickness and density; AA displayed a positive correlation with pyrogallol concentration; and mechanical properties such as TS and EAB were positively correlated, highlighting the increase of strength and flexibility from F1 to F4. Additionally, antibacterial activity against *S. aureus*, *K. pneumoniae*, and *E. coli* correlated positively with pyrogallol concentration. This analysis demonstrates that the structural integrity has been modified by adding potato starch and pyrogallol to the pectin film matrix and shows the hydrophilic, antibacterial, and antioxidant properties of pyrogallol.

### 3.18 Shelf‐life studies

#### 3.18.1 Weight loss rate

**FIGURE 8:**
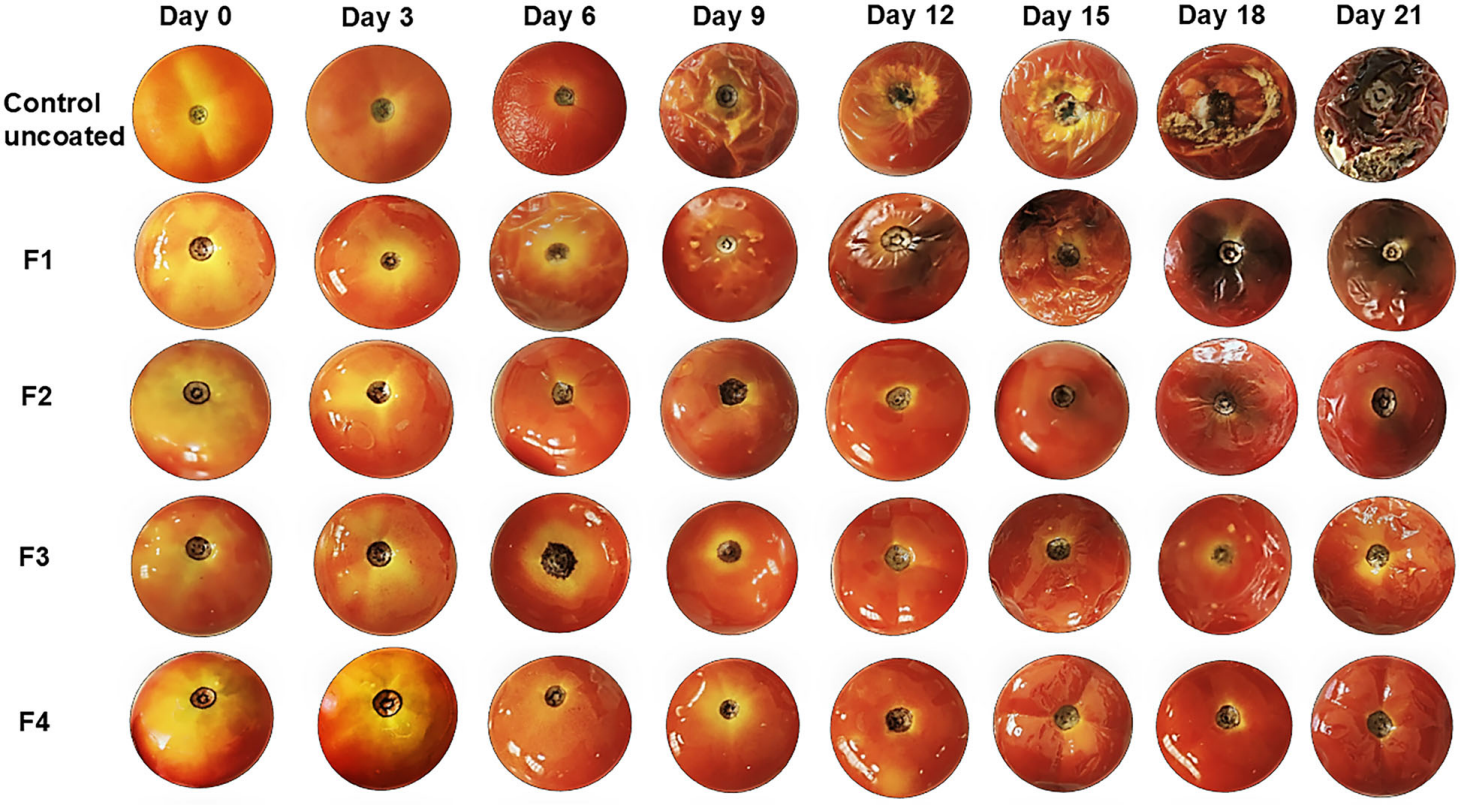
Effect of storage period (21 days) of tomatoes on uncoated (control) and coated with films (F1, pectin; F2, pectin + potato starch; F3, pectin + potato starch + 0.5%pyrogallol; and F4, pectin + potato starch + 1%pyrogallol) at room temperature.

Several changes are observed during postharvest storage and maturity of a fruit (tomato). One such significant change was the loss of weight. This was mainly due to the increase in the evaporation rate of water from the cells of tomatoes at the time of postharvest handling and storage, which leads to surface shrinking and textural weakening. On Days 0 and 3 of storage, no significant difference in the control and coated samples was observed. A slight change was visible from Day 6 onward, where the uncoated control sample had lost 4.1% of the weight (Table 5). This was due to the direct exposure of tomatoes to the environment. Due to the presence of a film (F1–F4) coating between the tomato fruit and the environment, the rate of evaporation of water, that is, the weight loss, was prevented. By the end of Day 21 (Figure 8), it was noted that the highest weight loss was observed in the uncoated (control) tomatoes (22.2%), followed by F1 (20.2%), F2 (18.7%), and F3 (16.9%), and the least weight loss was observed in F4 (14.5%). These results are in accordance with another study where strawberries packed with films containing chitosan‐thermoplastic starch material showed weight loss of up to 51.2% and 53.8% with 1% cinnamon and 1% ho wood essential oil, respectively (Ferreira et al., 2021). Another study comprising basil mucilage and cumin essential oil reported a weight loss of 19% on Day 9 (Tabarestani et al., 2013). Similarly, carboxymethylcellulose and aloe vera gel coating showed a weight loss of 20% on Day 20 (Kanmani et al., 2017).

**TABLE 5:** Shelf‐life studies of the developed coatings on tomatoes after a storage period of 21 days.

Films	Storage period (days)	Lycopene content (mg/100 g)	Total soluble solids (%)	pH	Titratable acidity (%)	Firmness (N)	Weight loss (%)	Total phenolic content (mg/100 g)	Antioxidant (%)
Control	0	0.32 ± 0.21^f^	3.1 ± 0.05^g^	3.39 ± 0.01^d^	0.66 ± 0.006^a^	99.2 ± 2.82^a^	0^h^	38.8 ± 0.33^a^	78 ± 0.5^a^
	3	0.6 ± 0.43^e^	3.6 ± 0.1^f^	3.47 ± 0.02^d^	0.62 ± 0.004^a^	93.5 ± 0.9^a^	1.2 ± 0.17^g^	35.7 ± 0.28^b^	72 ± 0.5^b^
	6	0.8 ± 0.21^e^	4 ± 0.05^e^	3.91 ± 0.02^c^	0.52 ± 0.003^b^	84.8 ± 2.68^b^	4.1 ± 0.42^f^	33.6 ± 0.33^c^	68 ± 1^c^
	9	1.14 ± 0.21^d^	4.4 ± 0.15^d^	4.39 ± 0.02^b^	0.47 ± 0.004^c^	70.3 ± 1.14^c^	7 ± 1.65^e^	30.8 ± 0.32^d^	61 ± 0.5^d^
	12	1.35 ± 0.21^d^	4.8 ± 0.05^d^	4.53 ± 0.02^b^	0.4 ± 0.004^d^	58.8 ± 1.05^d^	10 ± 1.85^d^	29.1 ± 0.51^e^	56 ± 1.5^e^
	15	1.82 ± 0.64^c^	5.3 ± 0.1^c^	4.66 ± 0.02^ab^	0.36 ± 0.002^e^	47.6 ± 0.78^e^	14.5 ± 1.03^c^	24.9 ± 0.32^f^	52 ± 1^f^
	18	2.17 ± 0.64^b^	5.8 ± 0.05^b^	4.75 ± 0.02^a^	0.31 ± 0.003^f^	33.6 ± 2.18^f^	18.6 ± 0.28^b^	22.4 ± 0.14^g^	48 ± 1^g^
	21	2.44 ± 0.43^a^	6.2 ± 0.05^a^	4.89 ± 0.02^a^	0.27 ± 0.002^g^	18.7 ± 2.73^g^	22.2 ± 0.48^a^	19.8 ± 0.32^h^	41 ± 0.5^h^
F1	0 3 6 9 12 15 18 21	0.28 ± 0.21^e^ 0.54 ± 0.21^d^ 0.71 ± 0.21^cd^ 1.05 ± 0.21^c^ 1.33 ± 0.43^c^ 1.72 ± 0.43^b^ 2.02 ± 0.43^a^ 2.25 ± 0.64^a^	3.2 ± 0.1^f^ 3.6 ± 0.05^e^ 3.9 ± 0.05^de^ 4.2 ± 0.15^d^ 4.6 ± 0.05^c^ 5.2 ± 0.1^b^ 5.5 ± 0.1^ab^ 5.9 ± 0.1^a^	3.37 ± 0.02^d^ 3.42 ± 0.01^d^ 3.86 ± 0.01^c^ 4.37 ± 0.02^b^ 4.55 ± 0.03^b^ 4.65 ± 0.03^ab^ 4.73 ± 0.01^a^ 4.85 ± 0.01^a^	0.67 ± 0.002^a^ 0.62 ± 0.003^a^ 0.53 ± 0.001^b^ 0.48 ± 0.002^c^ 0.42 ± 0.002^d^ 0.38 ± 0.001^e^ 0.34 ± 0.002^f^ 0.3 ± 0.004^g^	105.4 ± 0.77^a^ 97.3 ± 1.44^a^ 84.2 ± 2.26^b^ 72.8 ± 1.22^c^ 62.7 ± 2.04^d^ 53 ± 1.72^e^ 40.9 ± 1.69^f^ 30.7 ± 1.61^g^	0^h^ 1.2 ± 0.19^g^ 3.6 ± 0.31^f^ 6.3 ± 0.15^e^ 8.9 ± 0.23^d^ 12.3 ± 0.25^c^ 16.2 ± 0.38^b^ 20.2 ± 0.71^a^	39 ± 0.32^a^ 36 ± 0.23^b^ 33.9 ± 0.37^c^ 31.3 ± 0.33^d^ 29.5 ± 0.46^e^ 25.2 ± 0.33^f^ 23.2 ± 0.28^g^ 20.9 ± 0.42^h^	77 ± 0.5^a^ 73 ± 0.5^b^ 69 ± 0.5^c^ 64 ± 1^cd^ 58 ± 1^e^ 51 ± 0.5^f^ 47 ± 1.5^g^ 43 ± 1.5^g^
F2	0	0.32 ± 0.21^e^	3.1 ± 0.1^f^	3.28 ± 0.01^d^	0.65 ± 0.003^a^	106.5 ± 3.57^a^	0^h^	39.2 ± 0.23^a^	79 ± 1^a^
	3	0.49 ± 0.21^de^	3.5 ± 0.05^e^	3.41 ± 0.01^d^	0.62 ± 0.004^a^	97.2 ± 1.53^a^	1.1 ± 0.81^g^	36.3 ± 0.28^b^	74 ± 0.5^b^
	6	0.62 ± 0.21^d^	3.9 ± 0.1^de^	3.81 ± 0.01^c^	0.55 ± 0.004^b^	87 ± 1.62^b^	3.3 ± 1.12^f^	34.2 ± 0.28^c^	70 ± 0.5^bc^
	9	0.92 ± 0.21^cd^	4.2 ± 0.1^d^	4.37 ± 0.03^b^	0.49 ± 0.002^c^	75.9 ± 1.04^c^	5.8 ± 0.61^e^	31.6 ± 0.33^d^	65 ± 0.5^cd^
	12	1.2 ± 0.43^c^	4.4 ± 0.05^cd^	4.46 ± 0.01^b^	0.44 ± 0.001^d^	69.3 ± 0.9^c^	8.2 ± 0.25^d^	29.7 ± 0.38^e^	58 ± 0.5^e^
	15	1.61 ± 0.21^bc^	4.8 ± 0.05^c^	4.65 ± 0.01^ab^	0.4 ± 0.004^e^	57.4 ± 1.01^d^	12 ± 0.78^c^	26.1 ± 0.33^f^	52 ± 1^f^
	18	1.87 ± 0.64^a^	5.4 ± 0.1^b^	4.71 ± 0.02^a^	0.35 ± 0.004^f^	46.3 ± 1.39^e^	15.2 ± 0.4^b^	23.7 ± 0.37^g^	48 ± 1.5^g^
	21	2.06 ± 0.43^a^	5.8 ± 0.05^a^	4.81 ± 0.01^a^	0.31 ± 0.002^g^	36.9 ± 1.57^f^	18.7 ± 0.08^a^	21.6 ± 0.18^h^	44 ± 0.5^g^
F3	0	0.28 ± 0.21^e^	3.2 ± 0.1^f^	3.3 ± 0.02^d^	0.65 ± 0.003^a^	108.3 ± 1.17^a^	0^h^	39 ± 0.18^a^	79 ± 1^a^
	3	0.45 ± 0.21^de^	3.4 ± 0.1^ef^	3.4 ± 0.01^d^	0.63 ± 0.002^a^	98.2 ± 0.44^a^	1.2 ± 0.08^g^	36.5 ± 0.28^b^	75 ± 0.5^b^
	6	0.58 ± 0.21^d^	3.7 ± 0.05^e^	3.77 ± 0.02^c^	0.56 ± 0.002^b^	87.7 ± 1.27^b^	3.1 ± 0.41^f^	35 ± 0.18^b^	72 ± 0.5^bc^
	9	0.84 ± 0.21^cd^	4.1 ± 0.1^d^	4.23 ± 0.02^b^	0.51 ± 0.002^c^	78 ± 1.28^c^	4.5 ± 0.45^e^	32.5 ± 0.28^c^	67 ± 0.5^d^
	12	1.09 ± 0.21^c^	4.3 ± 0.05^cd^	4.34 ± 0.02^b^	0.46 ± 0.002^d^	65.4 ± 1.13^d^	6.6 ± 0.44^d^	30.4 ± 0.23^d^	60 ± 1^e^
	15	1.39 ± 0.64^c^	4.6 ± 0.05^cd^	4.53 ± 0.02^ab^	0.43 ± 0.002^e^	53.2 ± 1.42^e^	9.8 ± 0.03^c^	26.9 ± 0.18^e^	57 ± 0.5^ef^
	18	1.69 ± 0.64^b^	5.2 ± 0.1^b^	4.63 ± 0.01^ab^	0.38 ± 0.004^f^	46.3 ± 1.93^e^	13.3 ± 0.26^b^	24.9 ± 0.28^f^	54 ± 2^f^
	21	1.89 ± 0.43^a^	5.7 ± 0.1^a^	4.75 ± 0.02^a^	0.35 ± 0.002^f^	42.6 ± 1.37^f^	16.9 ± 0.08^a^	23 ± 0.23^g^	48 ± 1.5^g^
F4	0	0.3 ± 0.43^d^	3.1 ± 0.1^e^	3.29 ± 0.02^d^	0.66 ± 0.002^a^	107.2 ± 1.43^a^	0^h^	39 ± 0.28^a^	78 ± 0.5^a^
	3	0.41 ± 0.21^cd^	3.3 ± 0.05^de^	3.38 ± 0.01^d^	0.63 ± 0.002^a^	99.4 ± 0.58^a^	1.1 ± 0.05^g^	36.7 ± 0.19^b^	76 ± 0.5^ab^
	6	0.54 ± 0.21^cd^	3.6 ± 0.1^d^	3.7 ± 0.02^c^	0.56 ± 0.003^b^	88.5 ± 1.6^b^	3 ± 0.17^f^	35.4 ± 0.09^b^	74 ± 1^b^
	9	0.75 ± 0.21^c^	3.9 ± 0.05^cd^	4.13 ± 0.01^b^	0.53 ± 0.002^b^	80.2 ± 1.3^bc^	4.4 ± 0.09^e^	33 ± 0.18^c^	70 ± 0.5^bc^
	12	0.92 ± 0.21^bc^	4.1 ± 0.05^c^	4.25 ± 0.03^b^	0.49 ± 0.005^c^	70 ± 1.23^c^	5.8 ± 0.13^d^	31.8 ± 0.23^d^	65 ± 1^cd^
	15	1.22 ± 0.21^b^	4.4 ± 0.05^bc^	4.45 ± 0.02^ab^	0.46 ± 0.002^d^	62.5 ± 0.94^d^	7.6 ± 0.02^c^	29.5 ± 0.42^e^	60 ± 0.5^e^
	18	1.54 ± 0.43^a^	4.9 ± 0.1^b^	4.57 ± 0.02^ab^	0.42 ± 0.002^e^	52.8 ± 1.56^e^	11.4 ± 0.16^b^	27.6 ± 0.23^f^	57 ± 2^e^
	21	1.72 ± 0.43^a^	5.4 ± 0.1^a^	4.68 ± 0.02^a^	0.38 ± 0.003^f^	46 ± 1.62^e^	14.5 ± 0.12^a^	25 ± 0.23^g^	53 ± 1.5^f^

*Note*: Control, uncoated tomatoes; F1, pectin; F2, pectin + potato starch; F3, pectin + potato starch + 0.5%pyrogallol; and F4, pectin + potato starch + 1%pyrogallol. All data are averages of three measurements and reported as mean ± SD. According to the Bonferroni test, different letters (a, b, c, d, e, f, g, h) indicate significant differences between groups at *p* < 0.05.

#### 3.18.2 Total soluble solids (TSS)

Complex sugars are broken down into simple ones during the ripening process in the cell wall of fruits, which causes an increase in TSS, leading to the unripe fruits and vegetables’ ripe and overripe stages (Kumar et al., 2021). The longer the time taken for the TSS levels to increase, the better the food's shelf stability. It was observed that there was a steady increase in the TSS levels for all the tomatoes (both coated and uncoated). However, the uncoated tomatoes had higher TSS values than the coated tomatoes, and this increase in TSS demonstrates the faster maturity of the tomatoes. On Day 21, the lowest and desirable TSS levels were observed in F4 (5.4%), followed by F3 (5.7%); however, the highest and undesirable levels were observed in the control (uncoated) tomatoes (6.2%), followed by F1 (5.9%) and F2 (5.8%) (Table 5). The acceptable TSS limit for common or round tomatoes ranges from 3.5 to 5.5. In our study, F4‐coated tomatoes exhibited a TSS of 5.4% on Day 21, whereas control (uncoated) tomatoes reached the same TSS value around Day 15. This indicates that the F4 coating effectively delayed TSS accumulation, contributing to prolonged shelf life and maintaining fruit quality over an extended storage period (Yara, 2025). The coatings act as a semipermeable membrane on the fruit, and they produce and trap carbon dioxide gas inside, restricting the entry of oxygen. Other researchers also observed a similar trend, which suggests a reduction in metabolite synthesis and utilization, a decrease in respiration rate, and a repressed production of ethylene gas, which helps control fruit maturity. A similar increase in TSS (5.8%) was observed in the non‐coated tomatoes after a storage period of 15 days, whereas a 5.3% increase was observed in the tomatoes coated with xanthan gum, whey protein isolate, and clove oil (Kumar & Saini, 2021).

#### 3.18.3 Titratable acidity (TA) and pH

TA and pH are inversely proportional to each other (Table 5). A reduction in TA content was observed in the tomato samples. On Day 21, the least was observed in the uncoated tomatoes (0.27%), followed by F1 (0.3%), F2 (0.31%), and F3 (0.35%), and the highest was observed in F4 (0.38%). A similar decreasing trend in TA was observed for aloe vera and chitosan coatings applied to tomatoes (Farooq et al., 2023). Furthermore, another study comprising mango kernel starch coating showed a TA of 0.2% on Day 20 (Nawab et al., 2017). However, pH increases from Day 0 to 21 with the uncoated (control) sample at 4.89, followed by F1 (4.85), F2 (4.81), F3 (4.75), and the least in F4 (4.68). This suggests that adding pyrogallol has a noticeable effect on delaying the ripening process. TA decreases with the increase in storage period due to the conversion of organic acids to sugars, slowing down the metabolism of tomatoes. It changes the functional and structural properties of the films, thereby increasing the pH. A similar trend of an increase in pH was recorded in the case of nano‐fibrillated banana pseudo stem cellulose–polyvinyl alcohol–polyacrylic acid coatings applied on tomatoes (Ponni et al., 2020).

#### 3.18.4 Firmness

Firmness was a critical factor in selling fruit to a customer, and if the fruit was incredibly soft, it might be easily squished, thereby being rejected by the customer. All the tomatoes lost their firmness by Day 21 (Table 5), but the highest decrease in firmness was noticed in the uncoated (control) tomatoes, where the firmness reduced from 99.2 to 18.7 N. This was followed by F1 (105.4–30.7 N), F2 (106.5–36.9 N), F3 (108.3–42.6 N), and F4 (107.2–46 N) (Figure 8). Firmness depends upon the cell's turgor pressure, the strength of the cell wall, and the contact of one cell to the other (Zhou et al., 2021). Reduction in firmness suggests the degradation of cellular components and pectin due to decay of tissues and softening of tomatoes. This occurs due to hydrolytic enzymes, which are enhanced during storage as the tomatoes ripen. Moreover, water loss also enhances tomatoes’ softening. The results align with another study where the firmness of chitosan‐polyphenol‐silver coated tomatoes was 83% higher than the uncoated by the end of Day 20 (Huynh et al., 2022). Similarly, a study on mango kernel starch coating showed a firmness of 20 N on Day 20 (Nawab et al., 2017). Another study on guar gum reported a firmness of 15 N on Day 20 (Ruelas‐Chacon et al., 2017).

#### 3.18.5 Total phenolic content and antioxidant activity

The coatings of the film influence the phenolic oxidation, production of ethylene gas, and respiration rate, thereby increasing the shelf life. A consistent TPC reduction was noticed in the uncoated and coated tomato samples (Table 5). It was seen that the uncoated tomatoes did not show an increased amount of phenolic content than the coated samples. However, the coated films yielded comparatively more phenols. On Day 21, the uncoated control sample had the least phenolic content (19.8 mg/100 g), closely followed by F1 with 20.9 mg/100 g and F2 with 21.6 mg/100 g. However, F3 and F4 had comparatively more phenolic content, 23 and 25 mg/100 g, respectively. A similar trend of the preservation of the TPC was reported for chitosan–pullulan–pomegranate peel extract coatings on tomatoes (Kumar et al., 2021).

The active component pyrogallol scavenges the free radicals, and the AA was inversely related to the storage period; hence, by Day 21, the AA drastically reduced for all samples (Table 5). However, a significant difference was observed in the coated and uncoated samples. The uncoated tomatoes displayed the least AA (41%), followed by F1 (43%) and F2 (44%). However, with the addition of an antioxidant compound, pyrogallol, the AA observed was higher, 48% and 53% for F3 and F4, respectively, and this suggests that AA was retained better after 21 days. This protects the tomatoes from microbial contamination and gas exchange, thereby reducing lipid oxidation (Kumar et al., 2021). A similar result of retaining AA was observed in chitosan–pullulan–pomegranate peel extract‐based coatings on tomatoes, where AA was decreased from 50% to 26.1% (coated) and 50% to 18.1% (untreated) tomatoes in 15 days of storage (Kumar et al., 2021).

#### 3.18.6 Lycopene content

Lycopene is non‐provitamin A and a carotenoid responsible for fruits such as tomatoes’ pink to red color. Lycopene content was directly associated with storage and ripening; the longer the tomato was stored, the higher the lycopene content was recorded (Story et al., 2010). It was observed that on Day 21, the uncoated (control) tomatoes showed 2.44 mg/100 g; meanwhile, F4 displayed only 1.72 mg/100 g of lycopene, followed by F3 (1.89 mg/100 g), F2 (2.06 mg/100 g), and F1 (2.25 mg/100 g) (Table 5). This suggests that adding coatings delays the ripening and keeps the lycopene content at a reduced level (Dehghani et al., 2022). A decrease in lycopene content was observed for the tomatoes coated with rice bran wax during 21 days of storage (Abhirami et al., 2020).

#### 3.18.7 Color measurement

Ripening of tomatoes occurs due to the component chromoplasts, where chloroplasts are converted into carotenoid‐accumulating plastids. It provides tomatoes with yellow, orange, or red color to the ripening tomatoes (Ling et al., 2021). This study observed that the value of lightness (L*) decreases with the increase in storage period due to the transition of tomatoes from unripe to ripe. Similar results were observed in another study where different ripeness stages were determined (González‐Casado et al., 2018). As yellowness was measured by b* and redness by a*, this parameter estimates the degree of ripeness. It was observed that the rate of ripening was higher in uncoated (control) tomatoes as a* increased from 20.6 to 31.8 by Day 21 (Table 6) when compared to the coated fruits (F4, 20.6–29.9; F3, 20.6–30.7; F2, 20.6–31.4; and F1, 20.6–31.6). Meanwhile, b* decreased from 28.93 to 14.23 in uncoated fruits in comparison to the coated fruits (F4, 28.9–15.5; F3, 28.9–15.3; F2, 28.8–14.9; and F1, 28.9–14.7). A similar trend was observed for chitosan‐grapeseed extract‐based coatings on cherry tomatoes (Won et al., 2018). Values of a*/b* depicted the slower maturity rate of F4‐coated tomatoes, with the ratio increasing from 0.7 to 1.9, whereas a faster maturity rate was observed in uncoated tomatoes (0.7–2.2). This confirms that the uncoated tomatoes reach the ripening stage earlier than the coated tomatoes. Similar results of an increase in the ratio of a*/b* were observed, depicting the slower ripening of tomatoes when an edible coating made of chitosan and crude green algal ethanoic biomass extract was applied (Mondal et al., 2022). Another study on basil mucilage and cumin essential oil reported an a*/b* value of 1.2 on Day 9 (Tabarestani et al., 2013).

**TABLE 6:** Color analysis of the developed coatings on tomatoes after a storage period of 21 days.

Films	Storage period (days)	Color analysis			
		L*	a*	b*	Ratio of red and yellow (a*/b*)
Control	0	48.2 ± 0.3^a^	20.6 ± 0.03^f^	28.9 ± 0.02^a^	0.711 ± 0.0015^e^
	3	45.9 ± 0.28^b^	22.4 ± 0.04^e^	27.2 ± 0.01^ab^	0.821 ± 0.0013^de^
	6	42.3 ± 0.29^c^	23.8 ± 0.09^de^	25 ± 0.01^b^	0.949 ± 0.003^d^
	9	38.3 ± 0.29^d^	25.4 ± 0.04^c^	22.8 ± 0.03^c^	1.11 ± 0.0003^c^
	12	33.4 ± 0.36^e^	26.9 ± 0.05^c^	20.9 ± 0.04^cd^	1.286 ± 0.0001^bc^
	15	29 ± 0.25^f^	27.6 ± 0.05^bc^	18.6 ± 0.03^d^	1.482 ± 0.0002^b^
	18	25.7 ± 0.23^g^	29.7 ± 0.02^b^	16.1 ± 0.02^de^	1.844 ± 0.0013^b^
	21	21.4 ± 0.1^h^	31.8 ± 0.02^a^	14.2 ± 0.02^e^	2.234 ± 0.0025^a^
F1	0 3 6 9 12 15 18 21	48.5 ± 0.3^a^ 46.1 ± 0.1^b^ 42.6 ± 0.19^c^ 38.5 ± 0.3^d^ 33.8 ± 0.14^e^ 29.2 ± 0.24^f^ 26 ± 0.15^g^ 21.9 ± 0.16^h^	20.6 ± 0.04^f^ 22.3 ± 0.01^e^ 23.6 ± 0.03^de^ 25.3 ± 0.04^c^ 26.4 ± 0.03^c^ 27.3 ± 0.04^cd^ 29.4 ± 0.04^b^ 31.6 ± 0.02^a^	28.9 ± 0.02^a^ 27.3 ± 0.03^ab^ 25.6 ± 0.01^b^ 23.4 ± 0.02^c^ 21.1 ± 0.02^cd^ 18.9 ± 0.02^d^ 16.5 ± 0.03^de^ 14.7 ± 0.02^e^	0.713 ± 0.0009^e^ 0.815 ± 0.0005^de^ 0.924 ± 0.0008^d^ 1.078 ± 0.0008^dc^ 1.251 ± 0.0002^bc^ 1.441 ± 0.0002^b^ 1.783 ± 0.0008^b^ 2.142 ± 0.0019^a^
F2	0 3 6 9 12 15 18 21	48.4 ± 0.13^a^ 46.2 ± 0.11^b^ 42.9 ± 0.07^c^ 38.6 ± 0.11^d^ 34.2 ± 0.12^e^ 29.5 ± 0.42^f^ 26.3 ± 0.06^g^ 22.5 ± 0.22^h^	20.6 ± 0.03^f^ 22.3 ± 0.01^e^ 23.4 ± 0.04^de^ 25.1 ± 0.03^c^ 26.3 ± 0.03^c^ 27.2 ± 0.04^bc^ 29.3 ± 0.03^b^ 31.4 ± 0.03^a^	28.8 ± 0.03^a^ 27.5 ± 0.01^ab^ 25.7 ± 0.02^b^ 23.6 ± 0.03^c^ 22.6 ± 0.03^cd^ 21.3 ± 0.03^d^ 19 ± 0.02^de^ 14.9 ± 0.02^e^	0.713 ± 0.0019^e^ 0.811 ± 0.0001^de^ 0.91 ± 0.001^d^ 1.063 ± 0.0001^cd^ 1.165 ± 0.0^c^ 1.273 ± 0.0003^bc^ 1.539 ± 0.0002^b^ 2.109 ± 0.0015^a^
F3	0 3 6 9 12 15 18 21	48.4 ± 0.12^a^ 46.4 ± 0.13^b^ 43.2 ± 0.08^c^ 39.4 ± 0.12^d^ 35.2 ± 0.22^e^ 30.2 ± 0.09^f^ 27.5 ± 0.35^g^ 23.6 ± 0.12^h^	20.6 ± 0.02^f^ 21.9 ± 0.03^e^ 23.3 ± 0.03^ed^ 25 ± 0.02^c^ 25.8 ± 0.02^c^ 26.8 ± 0.01^bc^ 28.7 ± 0.02^b^ 30.7 ± 0.03^a^	28.9 ± 0.01^a^ 27.6 ± 0.02^ab^ 25.9 ± 0.02^b^ 24 ± 0.02^c^ 22.7 ± 0.02^cd^ 21.5 ± 0.01^d^ 19.6 ± 0.02^de^ 15.3 ± 0.03^e^	0.712 ± 0.0009^e^ 0.794 ± 0.0016^e^ 0.898 ± 0.0022^d^ 1.043 ± 0.0017^cd^ 1.135 ± 0.0021^c^ 1.248 ± 0.0016^bc^ 1.463 ± 0.0029^b^ 2.008 ± 0.0059^a^
F4	0 3 6 9 12 15 18 21	48.2 ± 0.17^a^ 46.8 ± 0.13^b^ 43.4 ± 0.16^c^ 40.3 ± 0.17^d^ 37.4 ± 0.12^e^ 31.6 ± 0.27^f^ 29.1 ± 0.19^g^ 25.3 ± 0.31^h^	20.6 ± 0.01^f^ 21.8 ± 0.02^e^ 23.1 ± 0.03^de^ 24.7 ± 0.03^c^ 25.5 ± 0.02^c^ 26.3 ± 0.03^bc^ 28.2 ± 0.02^b^ 29.9 ± 0.02^a^	28.9 ± 0.02^a^ 27.8 ± 0.02^ab^ 26 ± 0.02^b^ 24.2 ± 0.02^c^ 22.9 ± 0.02^cd^ 21.8 ± 0.01^d^ 19.9 ± 0.02^de^ 15.5 ± 0.03^e^	0.712 ± 0.0001^e^ 0.78 ± 0.0002^e^ 0.89 ± 0.0007^d^ 1.021 ± 0.0004^cd^ 1.111 ± 0.0001^c^ 1.211 ± 0.0005^bc^ 1.415 ± 0.0002^b^ 1.926 ± 0.0053^a^

*Note*: Control, uncoated tomatoes; F1, pectin; F2, pectin + potato starch; F3, pectin + potato starch + 0.5%pyrogallol; and F4, pectin + potato starch + 1%pyrogallol. All data are averages of three measurements and reported as mean ± SD. According to the Bonferroni test, different letters (a, b, c, d, e, f, g, and h) indicate significant differences between groups at *p* < 0.05.

#### 3.18.8 Microbial analysis

**FIGURE 9:**
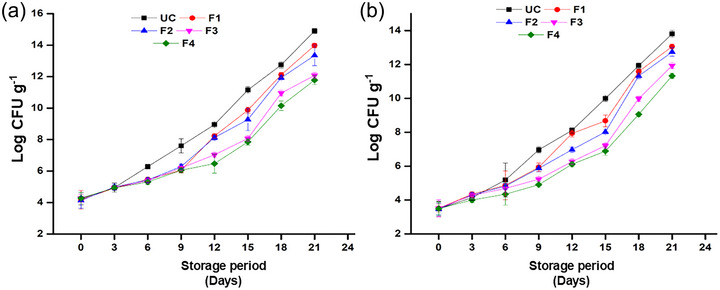
Microbial analysis (CFU count) of the uncoated and coated tomatoes during 21 days of storage (a) mesophilic bacterial count; (b) yeast and mold. F1, pectin; F2, pectin + potato starch; F3, pectin + potato starch + 0.5%pyrogallol; and F4, pectin + potato starch + 1%pyrogallol; UC, uncoated.

The microbial analysis demonstrated the infestation of microorganisms in the tomatoes throughout the storage period. It was observed that there was a steady increase in the microbial count for both coated and uncoated tomatoes during 21 days of storage. However, the coating combination of F4 on tomatoes showed the best results in controlling microbial growth. The uncoated tomatoes have 14.9 CFU/g mesophilic bacterial count and 13.8 CFU/g mold and yeast count; however, the microbial growth was restricted to 11.8 CFU/g mesophilic bacterial count and 11.3 CFU/g mold and yeast count (Figure 9). This was due to the presence of pyrogallol in the coating formulation. Pyrogallol has been widely reported to be an antimicrobial agent targeting cell integrity and survival. Hence, the restricted growth of microorganisms in the fruit enhances the shelf life of tomatoes (Tian et al., 2023). A similar trend was observed when the antimicrobial extract of *Flourensia cernua* coatings was applied to extend the shelf life of tomatoes (Salas‐Méndez et al., 2019). According to Hazard Analysis and Critical Control Point—Total Quality Management technical guidelines, more than 7.7 log CFU/g is considered spoiled food (Khadka et al., 2017). The uncoated, F1, and F2 tomatoes had a shelf life of 9 days (7.6, 6.4, and 6.3 log CFU/g, respectively). Meanwhile, F3 had a shelf life of 12 days (7.03 log CFU/g) and F4 almost 15 days (7.8 log CFU/g).

## 4 CONCLUSION

The current study investigates the development and properties of an active biodegradable film made from pectin, potato starch, and pyrogallol. The formulation F4 (pectin + potato starch + 1%pyrogallol) demonstrated superior antimicrobial (42 mm against *S. aureus*, 20.5 mm against *K. pneumoniae*, and 25.5 mm against *E. coli*), antioxidant (95%), mechanical, and biodegradation properties, highlighting each component's contribution to the film's overall quality. The application of this film successfully prevented signs of spoilage and significantly enhanced the shelf life of tomatoes (on Day 21, the firmness was observed to be 46 N, 14.5% weight loss, 1.7% lycopene content, and 53.5% AA). This research provides valuable insights into developing sustainable packaging materials containing pyrogallol, offering promising alternatives to traditional petroleum‐based packaging materials. By advancing the field of biodegradable films, this study provides an eco‐friendly and effective solution for food preservation and packaging. Future research will aim to refine coating formulations, investigate broader applications, study the effects of long‐term storage, assess consumer acceptance, and evaluate the environmental impact.

## AUTHOR CONTRIBUTIONS

**Aparna Ramadoss**: Methodology; data curation; validation; writing—original draft; writing—review and editing. **Venkata Giridhar Poosarla**: Conceptualization; methodology; data curation; investigation; validation; supervision; funding acquisition; visualization; project administration; resources; writing—original draft; writing—review and editing. **Shaik Sadiya**: Methodology; data curation; validation. **Nagaveni Shivshetty**: Methodology; data curation; validation; writing—original draft; writing—review and editing.

## CONFLICT OF INTEREST STATEMENT

The authors declare that there are no conflicts of interest.

## Supporting information



Supporting Information

## Data Availability

Data will be made available on request.
