# Supplementary material for: A novel active biopolymer coating of pectin, potato starch, and pyrogallol: Impact on postharvest quality of tomato (Solanum lycopersicum L.)
Source: J Food Sci. 2025 Apr 4;90(4):e70179. doi: 10.1111/1750-3841.70179 (PMC11970456; doi:10.1111/1750-3841.70179)
Supplement: Supplementary file 1 — Supporting Information [file JFDS-90-0-s001.docx]

**Supporting Information**

**A Novel Active Biopolymer Coating of Pectin, Potato Starch, and Pyrogallol: Impact on Postharvest Quality of Tomato (*Solanum lycopersicum* L.)**

**Names and e-mail addresses for all authors**

Author 1 Aparna Ramadoss aramados2@gitam.in

Author 2 Venkata Giridhar Poosarla gpoosarl@gitam.edu

Author 3 Shaik Sadiya ssadiya2@gitam.in

Author 4 Nagaveni Shivshetty nshivshe@gitam.edu

**Author affiliation(s)**

**Authors 1,2,3,4** Department of Life Sciences, GITAM School of Science, GITAM (Deemed to be University), Visakhapatnam, Andhra Pradesh, India, 530045

**Contact information for Corresponding author**

Venkata Giridhar Poosarla, Department of Life Sciences, GITAM School of Science, GITAM (Deemed to be University), Visakhapatnam, Andhra Pradesh, India, 530045

Email: gpoosarl@gitam.edu, Phone: +91-0891-2840472

**Short version of title –** Study of tomato shelf life with active coatings

**Choice of journal/topic** – Integrated Food Science

**1. Supporting Figures**


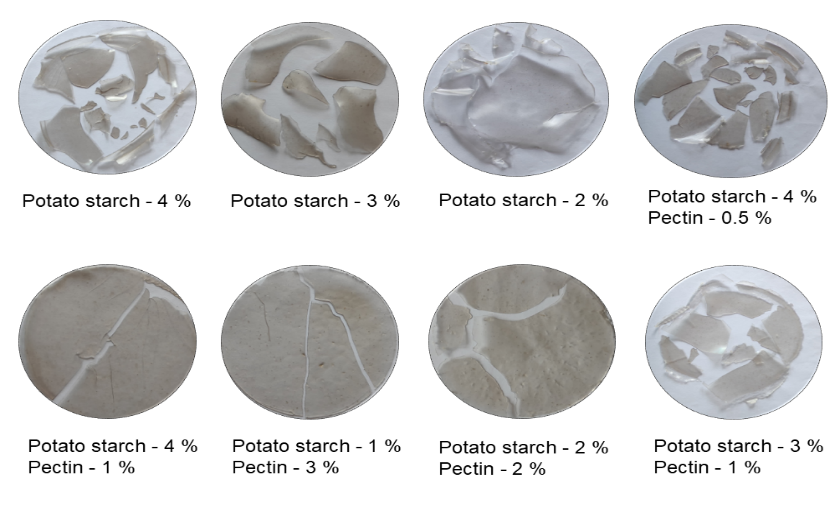


**Figure S1.** Testing of different combinations of potato starch and pectin for the selection of base composition for the formation of films.


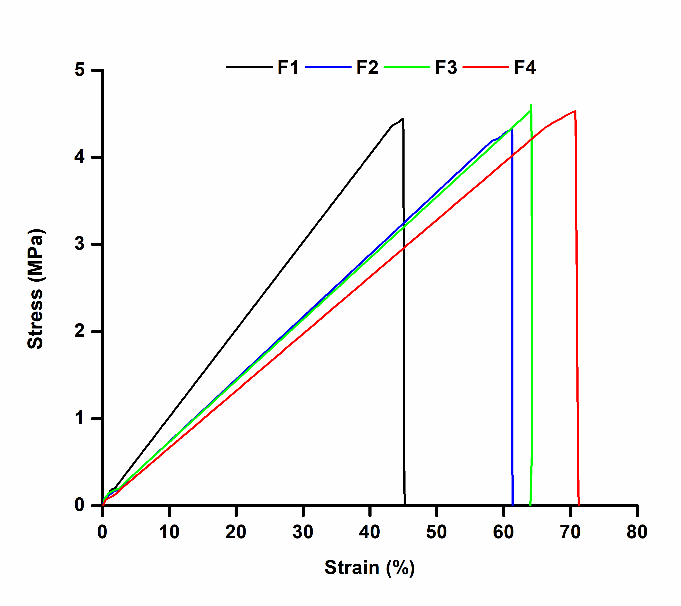


**Figure S2.** Stress-strain curves of the developed films F1-F4

**Note:** F1, Pectin; F2, Pectin+Potato starch; F3, Pectin+Potato starch+0.5%Pyrogallol; and F4, Pectin+Potato starch+1%Pyrogallol.


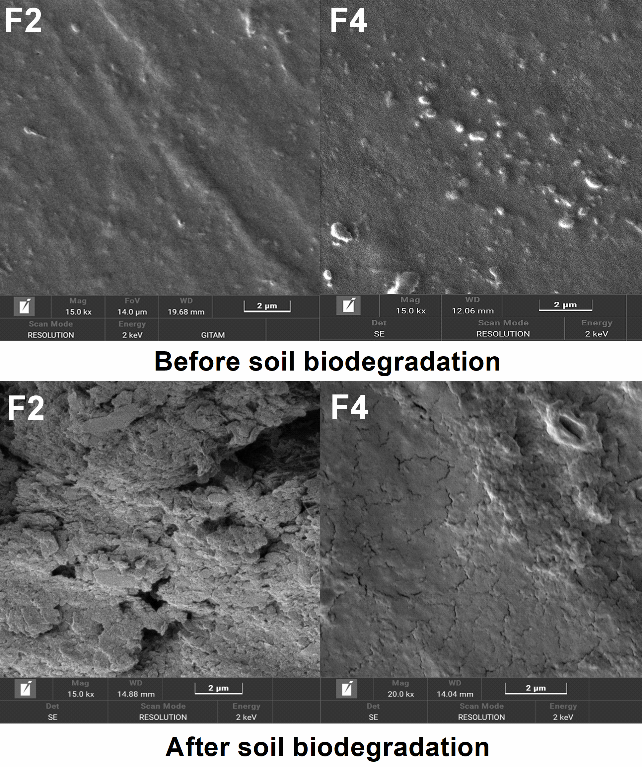


**Figure S3.** FE-SEM images of soil biodegradation of F2 and F4 films (before soil biodegradation (day 0) and after soil biodegradation (day 15)). F2, Pectin+Potato starch and F4, Pectin+Potato starch+1%Pyrogallol.

**2. Supporting Tables**

**Table S1.** Measurement of the shrinkage parameters of the developed films

| **Films** | **Machine direction (MD)** | **Transverse direction (TD)** |
| --- | --- | --- |
| F1 | 14±1^d^ | 16±1^d^ |
| F2 | 16±1^c^ | 19±1^c^ |
| F3 | 21±1^b^ | 24±1^b^ |
| F4 | 29±1^a^ | 27±2^a^ |

**Note:** F1, Pectin; F2, Pectin+Potato starch; F3, Pectin+Potato starch+0.5%Pyrogallol; and F4, Pectin+Potato starch+1%Pyrogallol.

**Table S2.** Pearson correlation for the possible correlations between treatments and/or variables of film

|  |  | **T (mm)** | **MC (%)** | **WS (%)** | **WVP (10^−11^ g/(m s Pa))** | **D (g cm^-3^)** | **WA** | **Ty (%)** | **O (A/mm)** | **Antioxidant activity (%)** | | | **Mechanical properties** | | | **Antibacterial**  **Activity (mm)** | | | **Film color** | | | | |
| --- | --- | --- | --- | --- | --- | --- | --- | --- | --- | --- | --- | --- | --- | --- | --- | --- | --- | --- | --- | --- | --- | --- | --- |
|  |  |  |  |  |  |  |  |  |  | **DPPH** | **HRSA** | **MCA** | **TS (MPa)** | **EAB (%)** | **YM (MPa)** | ***S. aureus*** | ***K. pneumoniae*** | ***E. coli*** | **L** | **a** | **b** | **ΔE (%)** | **WI** |
|  | **T (mm)** | 1 |  |  |  |  |  |  |  |  |  |  |  |  |  |  |  |  |  |  |  |  |  |
|  | **MC (%)** | -0.95 | 1.00 |  |  |  |  |  |  |  |  |  |  |  |  |  |  |  |  |  |  |  |  |
|  | **WS (%)** | -0.91 | 0.89 | 1.00 |  |  |  |  |  |  |  |  |  |  |  |  |  |  |  |  |  |  |  |
|  | **WVP (10^−11^ g/(m s Pa))** | 0.99 | -0.98 | -0.89 | 1.00 |  |  |  |  |  |  |  |  |  |  |  |  |  |  |  |  |  |  |
|  | **D (g cm^-3^)** | 1.00 | -0.94 | -0.92 | 0.99 | 1.00 |  |  |  |  |  |  |  |  |  |  |  |  |  |  |  |  |  |
|  | **WA** | 0.96 | -0.97 | -0.82 | 0.99 | 0.95 | 1.00 |  |  |  |  |  |  |  |  |  |  |  |  |  |  |  |  |
|  | **Ty (%)** | -0.91 | 1.00 | 0.87 | -0.95 | -0.91 | -0.96 | 1.00 |  |  |  |  |  |  |  |  |  |  |  |  |  |  |  |
|  | **O (A/mm)** | 0.88 | -0.98 | -0.79 | 0.93 | 0.87 | 0.96 | -0.99 | 1.00 |  |  |  |  |  |  |  |  |  |  |  |  |  |  |
| **Antioxidant Activity (%)** | **DPPH** | 0.87 | -0.96 | -0.74 | 0.92 | 0.85 | 0.97 | -0.97 | 0.99 | 1.00 |  |  |  |  |  |  |  |  |  |  |  |  |  |
|  | **HRSA** | 0.95 | -0.98 | -0.81 | 0.98 | 0.94 | 1.00 | -0.97 | 0.98 | 0.98 | 1.00 |  |  |  |  |  |  |  |  |  |  |  |  |
|  | **MCA** | 0.95 | -0.98 | -0.81 | 0.98 | 0.94 | 1.00 | -0.97 | 0.98 | 0.98 | 1.00 | 1.00 |  |  |  |  |  |  |  |  |  |  |  |
| **Mechanical properties** | **TS (MPa)** | 0.73 | -0.91 | -0.74 | 0.79 | 0.72 | 0.83 | -0.94 | 0.95 | 0.92 | 0.86 | 0.86 | 1.00 |  |  |  |  |  |  |  |  |  |  |
|  | **EAB (%)** | 0.84 | -0.91 | -0.97 | 0.85 | 0.85 | 0.80 | -0.91 | 0.85 | 0.79 | 0.82 | 0.81 | 0.86 | 1.00 |  |  |  |  |  |  |  |  |  |
|  | **YM (MPa)** | -0.90 | 0.98 | 0.78 | -0.95 | -0.89 | -0.98 | 0.98 | -1.00 | -1.00 | -0.99 | -0.99 | -0.92 | -0.82 | 1.00 |  |  |  |  |  |  |  |  |
| **Antibacterial**  **Activity (mm)** | ***S. aureus*** | 0.88 | -0.96 | -0.73 | 0.93 | 0.87 | 0.98 | -0.97 | 0.99 | 1.00 | 0.99 | 0.99 | 0.90 | 0.77 | -1.00 | 1.00 |  |  |  |  |  |  |  |
|  | ***K. pneumoniae*** | 0.99 | -0.95 | -0.85 | 0.99 | 0.99 | 0.98 | -0.92 | 0.90 | 0.91 | 0.97 | 0.97 | 0.74 | 0.80 | -0.93 | 0.92 | 1.00 |  |  |  |  |  |  |
|  | ***E. coli*** | 0.92 | -0.97 | -0.77 | 0.96 | 0.91 | 0.99 | -0.97 | 0.98 | 0.99 | 1.00 | 1.00 | 0.87 | 0.79 | -1.00 | 0.99 | 0.96 | 1.00 |  |  |  |  |  |
| **Film color** | **L** | -0.95 | 1.00 | 0.91 | -0.97 | -0.95 | -0.97 | 0.99 | -0.97 | -0.95 | -0.97 | -0.97 | -0.91 | -0.93 | 0.97 | -0.95 | -0.95 | -0.96 | 1.00 |  |  |  |  |
|  | **a** | 0.92 | -0.98 | -0.80 | 0.96 | 0.91 | 0.99 | -0.99 | 0.99 | 0.99 | 1.00 | 0.99 | 0.91 | 0.83 | -1.00 | 0.99 | 0.95 | 1.00 | -0.98 | 1.00 |  |  |  |
|  | **b** | 0.88 | -0.98 | -0.82 | 0.93 | 0.87 | 0.95 | -1.00 | 1.00 | 0.99 | 0.97 | 0.97 | 0.96 | 0.88 | -0.99 | 0.98 | 0.90 | 0.97 | -0.98 | 0.99 | 1.00 |  |  |
|  | **ΔE (%)** | 0.99 | -0.98 | -0.90 | 1.00 | 0.99 | 0.98 | -0.96 | 0.94 | 0.93 | 0.98 | 0.98 | 0.82 | 0.88 | -0.95 | 0.94 | 0.99 | 0.96 | -0.98 | 0.97 | 0.94 | 1.00 |  |
|  | **WI** | -0.94 | 1.00 | 0.90 | -0.97 | -0.94 | -0.97 | 1.00 | -0.98 | -0.96 | -0.98 | -0.98 | -0.91 | -0.92 | 0.98 | -0.96 | -0.94 | -0.97 | 1.00 | -0.98 | -0.99 | -0.98 | 1.00 |

**T**: Thickness; **MC**: Moisture content (%); **WS**: Water solubility (%); **SI**: Swelling index; **WVP**: Water vapor permeability (10^−11^ g/(m s Pa)); **D**: Density (g cm^−3^ ); **WA**: Water activity; **Ty**: Transparency (%); **O**: Opacity (A/mm); **AA**: Antioxidant Activity (%); **DPPH**: Diphenylpicrylhydrazyl assay; **HAS**: Hydroxyl radical-scavenging activity; **MCA**: Metal chelating activity; **TS**: Tensile strength (MPa); **EAB**: Elongation at break (%); **YM**: Youngs modulus (MPa); **ΔE**: Color difference (%); **WI**: Whiteness index.
